# The multifaceted roles of the ACSL family in cancer: Metabolic reprogramming, ferroptosis regulation and tumour immune microenvironment remodelling

**DOI:** 10.1002/ctm2.70643

**Published:** 2026-03-17

**Authors:** Haocai Li, Weijian Wang, Juncheng Zhan, Yuxiang Xiao, Xiaoping Chen, Chen Su, Peng Zhu

**Affiliations:** ^1^ Hepatic Surgery Center, Clinical Medical Research Center of Hepatic Surgery at Hubei Province Tongji Hospital, Tongji Medical College, Huazhong University of Science and Technology Wuhan China; ^2^ Hubei Key Laboratory of Hepato‐Pancreatic‐Biliary Diseases Tongji Hospital, Tongji Medical College, Huazhong University of Science and Technology Wuhan China; ^3^ Key Laboratory of Organ Transplantation Ministry of Education, Key Laboratory of Organ Transplantation, National Health Commission, and Key Laboratory of Organ Transplantation, Chinese Academy of Medical Sciences Wuhan China

**Keywords:** acyl‐CoA synthetase long‐chain family, ferroptosis, targeting therapy, tumour immunology, tumour metabolic reprogramming

## Abstract

**Background:**

Metabolic reprogramming is a key cancer hallmark, with dysregulated fatty acid metabolism critical for tumorigenesis and progression. The acyl‐CoA synthetase long‐chain (ACSL) family (ACSL1–ACSL6) catalyzes ATP‐dependent activation of long‐chain fatty acids into acyl‐CoA, a bioactive intermediate in lipid synthesis, β‐oxidation, membrane biogenesis, and signal transduction. Dysregulated ACSL expression is widespread in malignancies, but their non‐metabolic functions (ferroptosis regulation, tumor immune microenvironment remodeling) and translational potential of targeted therapies remain to be systematically summarized.

**Methods:**

This narrative review comprehensively synthesizes existing literature on the biological functions of ACSL family members in cancer. We retrieved and analyzed studies focusing on ACSL‐mediated lipid metabolic reprogramming, ferroptosis modulation, and immunomodulatory effects, with particular emphasis on isoform‐specific mechanisms and the context‐dependent roles (pro‐tumorigenic or tumor‐suppressive) of the ACSL family across different cancer types. Additionally, we summarized emerging therapeutic strategies targeting ACSL isoforms and their translational potential.

**Results:**

ACSL isoforms exert distinct context‐dependent effects:ACSL1 promotes immunosuppressive TIME via M2 macrophage polarization;ACSL3/4 have antagonistic roles in ferroptosis;ACSL5 exerts dual effects via lipid metabolism, apoptosis, and immunity;ACSL6 involves autophagy and hematological malignancies. Dysregulation correlates with tumor progression, drug resistance, and immunotherapy response, while emerging ACSL‐targeted drugs show substantial translational potential.

**Conclusions:**

The ACSL family serves as a key regulatory node integrating lipid metabolism, ferroptosis, and tumor immunity. Its isoform‐specific mechanisms and context‐dependent characteristics highlight its potential as a precise therapeutic target. Future research should focus on optimizing isoform‐selective inhibitors, clarifying their synergistic effects with existing therapies (e.g., immune checkpoint inhibitors, radiotherapy), and validating their translational efficacy through clinical trials to advance the development of innovative cancer treatment strategies.

## Key points

- Outlined the regulatory effects of the acyl‐CoA synthetase long‐chain (ACSL) family in different types of tumours.
- In‐depth analysis of the various functions of ACSL family members in tumour metabolic reprogramming, ferroptosis and tumour immunity.
- Revealed the intervention mechanism of emerging drugs targeting the ACSL family.
- Analysed the shortcomings and prospects of new drugs targeting the ACSL family.

## Graphical abstract

The acyl‐CoA synthetase long‐chain (ACSL) family consists of five members, which exhibit a synergistic effect in promoting tumour lipid metabolism for energy supply. Specifically, ACSL3 and ACSL4 have antagonistic effects on the mechanisms related to ferroptosis in tumour cells. Furthermore, each member of the ACSL family influences various immune cells within different tumour immune microenvironments. Currently, small molecule inhibitors that have been developed to inhibit members of the ACSL family show promising application prospects.

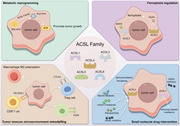

## 1 INTRODUCTION

In the 21st century, cancer remains a major global public health challenge.^1^ In 2022, cancer‐related deaths accounted for 16.8% of total mortality worldwide, ranking among the top three causes of death in individuals aged 30–69 years.^1^ While data from the American Cancer Society and National Health Institutes show an overall decline in cancer mortality over the past 5 years, the incidence of common malignancies (breast, pancreatic, prostate, liver, cervical, colorectal cancer [CRC] and melanoma) continues to increase annually.^2^, ^3^ Metabolic reprogramming, a hallmark of malignancy,^4^, ^5^ is characterised by dysregulated fatty acid metabolism that fuels tumour progression through enhanced lipid synthesis, energy supply and signal transduction.^6^, ^7^, ^8^, ^9^ This process has garnered increasing attention in cancer research. The acyl‐CoA synthetase long‐chain (ACSL) family, comprising five members (ACSL1, ACSL3, ACSL4, ACSL5 and ACSL6), plays a pivotal role in fatty acid activation.^10^ By governing acyl‐CoA production, ACSL members participate in tumour metabolic reprogramming and exhibit aberrant expression across the aforementioned malignancies, which is significantly correlated with clinical prognosis.^11^, ^12^, ^13^, ^14^, ^15^, ^16^, ^17^ In addition to their role in oncogenesis, ACSL enzymes also contribute to non‐neoplastic diseases such as ischemia reperfusion injury, myocardial infarction, osteoarthritis, diabetes and atherosclerosis.^18^, ^19^, ^20^, ^21^, ^22^, ^23^ Recent studies have expanded beyond elucidating ACSL‐driven metabolic rewiring to uncover their dual roles in ferroptosis regulation. For example, ACSL3 suppresses lipid peroxidation, whereas ACSL4 promotes ferroptosis, highlighting context‐dependent mechanisms.^24^, ^25^ Furthermore, ACSL isoforms reshape the heterogeneity of the tumour immune microenvironment (TIME) by modulating macrophage polarisation, T‐cell infiltration and immune checkpoint expression, thereby facilitating immune evasion.^26^, ^27^, ^28^ These discoveries have led to the development of innovative therapeutic strategies, including combinatorial approaches pairing ACSL inhibitors with pro‐ferroptotic agents (e.g., erastin)^29^ or immune checkpoint blockers.^30^

**FIGURE 1:**
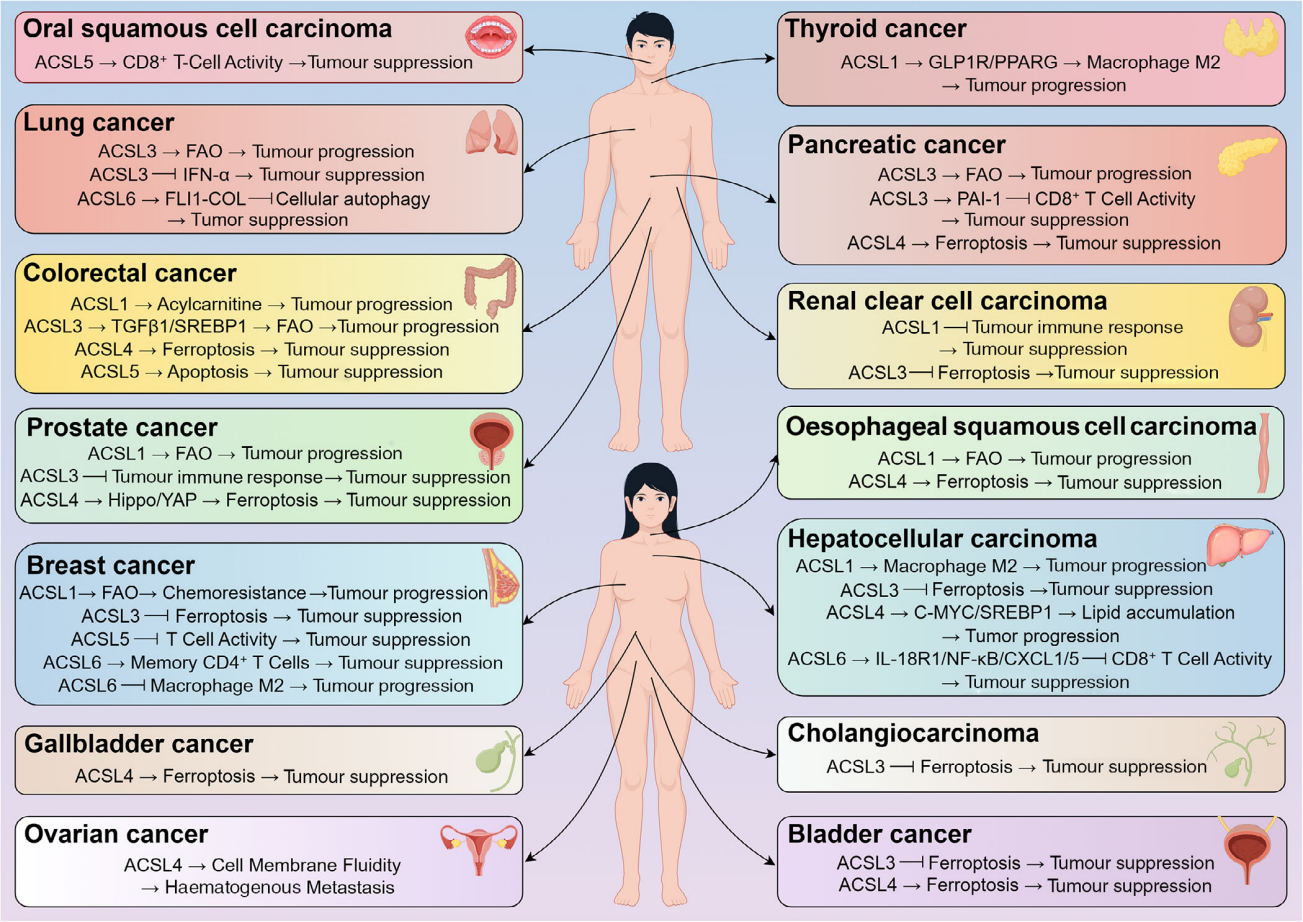
The regulatory mechanisms of acyl‐CoA synthetase long‐chain (ACSL) family in different types of tumours. The expression of ACSL family members is heterogeneous in different types of tumours, and the mechanisms regulating tumour occurrence and development are also different.

This review systematically dissects the mechanistic heterogeneity of ACSL family members in tumour metabolic reprogramming, ferroptosis regulation and immune modulation. This study further explores actionable interventions targeting ACSL enzymes, aiming to provide a theoretical foundation for developing precision therapies against cancer (Figure 1 and Table 1).

**TABLE 1:** The regulatory mechanisms and effects of ACSL family members in different types of tumours.

ACSL family members	Primary sites of expression	Function	Regulatory mechanism	Cancer type	Citations
•ACSL1	Heart, liver, adipose tissue and skeletal muscle	Promote tumourigenesis and development	Enhance lipid metabolism for energy supply	HCC, OC, PCa, ESCC	^38^, ^39^, ^40^, ^41^, ^43^
		Promote tumour invasion	Acylcarnitine	CRC	^46^
		Promote tumour immune escape	M2 polarisation of macrophages↑	PCa	^26^
		Impair T‐cell function	MAPK/NF‐κB pathway	BC	^50^
		Block T‐cell infiltration	CD44^+^ neutrophilsa	GC	^53^
		Foster immunosuppressive microenvironment	PD‐1, CTLA4↑	RCCC, LGG	^28^, ^54^
•ACSL3	Liver, pancreas, prostate, myocardial cells, mammary epithelial cells, vascular endothelial cells	Promote tumourigenesis and development	Enhance lipid metabolism for energy supply	HCC, PC, LC, TNBC	^11^, ^14^, ^65^, ^66^, ^67^
		Promote lymph node metastasis	ACSL3t	PDAC	^76^, ^77^
		Promote EMT and metastasis	TGFβ1/SREBP1 axis	CRC	^68^
		Drive CRPC growth	Promote intratumoural steroidogenesis	PCa	^69^, ^70^, ^71^, ^72^
		Promote proliferation and tumour formation	Akt pathway	MG	^75^
		Foster immunosuppressive microenvironment	TGF‐β/IL‐18 pathway	PCa	^71^, ^72^, ^88^
		Recruit Treg cells	LPCAT4/WNT/β‐catenin/c‐JUN pathway	HCC	^95^
		Inhibit ferroptosis	Reduce lipid peroxidation	BC, RCCC, MEL, OSCC, CRC, LUAD	^80^, ^81^, ^82^, ^83^, ^84^, ^85^, ^86^, ^87^
•ACSL4	Adrenal cortex, liver, brain microglia and reproductive system	Promote tumourigenesis and development	Enhance lipid metabolism for energy supply	HCC, BC	^117^, ^118^
		Promote haematogenous metastasis	Increase membrane fluidity and invasiveness	OC	^124^
		Promote proliferation and migration	TGF‐β/Smad2 pathway	OS	^119^
		Cause platinum resistance	Promote lipid raft formation	MEL	^16^
		Enhance anti‐tumour immunity	Promote dendritic cell maturation	AML	^139^
		Improve ICI efficacy	Promote IFN‐γ release from CD8^+^ T cells	LC	^140^
		Inhibit tumour growth	Promote lipid peroxidation and ferroptosis	CRC, PCa, GBC	^125^, ^126^, ^127^, ^128^, ^131^, ^132^, ^133^
•ACSL5	Intestine, liver, kidney and immune cells	Promote tumourigenesis and development	Enhance lipid metabolism for energy supply	BC	^151^
		Promote tumour cell apoptosis	HSPA9	CRC	^152^, ^153^
		Inhibit tumour proliferation	Wnt pathway↓	IC	^154^, ^155^
		Inhibit proliferation and migration	ERK pathway + enhance MHC‐I antigen presentation	LC	^156^, ^157^
		Enhance PD‐1 blockade efficacy	CD8^+^ T cell cytotoxicity/infiltratione	LC, OSCC, CM, OS	^158^, ^159^, ^160^, ^161^
		Reduce immunotherapy response	CD8^+^ T‐cell infiltrationapy responsetio	BC, MEL	^27^, ^164^
•ACSL6	Brain tissue, bone marrow and dopaminergic neurons	Cause recurrence and poor prognosis	Chromosomal translocation + IL‐3↑ + eosinophil expansion	AML, ALL	^169^, ^171^, ^173^, ^174^
		Reduce immunotherapy sensitivity	CD160/IFN‐γ pathway dysregulation	AML	^183^
		Inhibit tumour progression	Promote ferroptosis	PCa	^172^
		Promote immune escape	Recruit TAN/TAM + suppress CD8^+^ T‐cell infiltration	HCC	^184^
		Reduce ICI efficacy	PD‐1/CTLA4 via NF‐κB pathway	CRC	^185^
		Improve immunotherapy efficacy	Memory CD4^+^ T‐cell infiltration↑ + M2 macrophage↓	TNBC	^182^

Each entry corresponds to a specific regulatory mechanism validated by the cited studies. Citations are core references directly supporting the ‘regulatory mechanism’ and ‘cancer type’ pairing.

## 2 ACSL1

Acyl‐CoA synthetase long‐chain 1 (ACSL1), a pivotal enzyme in fatty acid metabolism.^31^, ^32^, ^33^ According to the NCBI and UNIPROT databases, ACSL1 is highly expressed in human liver, adipose tissue, heart, skeletal muscle and nervous system. Its subcellular localisation varies, encompassing mitochondria, the endoplasmic reticulum (ER) and the plasma membrane. Mitochondrial ACSL1 directly participates in fatty acid β‐oxidation (FAO) by catalysing the ATP‐dependent conversion of long‐chain fatty acids into acyl‐CoA, thereby regulating energy metabolism.^34^, ^35^ ACSL1 not only facilitates fatty acid uptake and activation but also enhances thermogenic capacity in adipocytes via the AMP‐activated protein kinase (AMPK)/peroxisome proliferator‐activated receptor gamma coactivator 1α (PGC1α) signalling axis. In hepatocytes, ACSL1 synergises with fatty acid translocase (FAT/CD36) to optimise fatty acid oxidation (FAO) efficiency and mitigate lipid accumulation.^31^, ^34^, ^35^ In addition to metabolic regulation, ACSL1 exerts transcriptional control by binding to the promoter region of the family with sequence similarity 13 member A gene (FAM13A), activating its expression to modulate lipid deposition.^36^ Nutritional status dynamically regulates ACSL1 expression: high‐fat diets or fasting downregulate ACSL1 mRNA levels, whereas protein abundance is governed by post‐translational modifications, underscoring its role in metabolic adaptation.^32^, ^37^ Overall, ACSL1 serves as a central orchestrator of lipid homeostasis and energy balance through metabolic reprogramming, energy sensing and gene regulatory mechanisms.

### 2.1 ACSL1 drives tumourigenesis via metabolic reprogramming

Lipid metabolic reprogramming orchestrated by acyl‐CoA synthetase long‐chain family member 1 (ACSL1) serves as a fundamental hallmark of oncogenesis across diverse malignancies through its regulation of bioenergetic and biosynthetic pathways. By enhancing mitochondrial FAO and acyl‐CoA synthesis, ACSL1 fuels ATP production for energy‐intensive processes while providing lipid precursors for membrane biogenesis and post‐translational modifications, driving biomass accumulation in proliferating tumour cells. Similarly, ovarian cancer hijacks ACSL1‐mediated protein myristoylation to activate AMPK/steroid receptor coactivator (Src) signalling cascades, amplifying FAO efficiency and facilitating metastatic dissemination through cytoskeletal remodelling.^38^, ^39^

Epigenetic and post‐translational regulatory mechanisms further modulate the oncogenic activity of ACSL1. In hepatocellular carcinoma (HCC), the tumour‐suppressive microRNA miR‐205 directly targets the 3′ untranslated region (3′UTR) of ACSL1 mRNA, downregulating its expression to impair lipid metabolic dysregulation and suppress tumour growth by restoring redox homeostasis.^40^ Conversely, oesophageal squamous cell carcinoma (ESCC) is characterised by SUMO‐specific peptidase 2 (SENP2)‐mediated de‐sumoylation of the SET domain bifurcated histone lysine methyltransferase 1 (SETDB1), which epigenetically upregulates ACSL1 transcription. This enhances fatty acid uptake and mitochondrial oxidation to accelerate proliferation through metabolic flux rewiring.^41^

**FIGURE 2:**
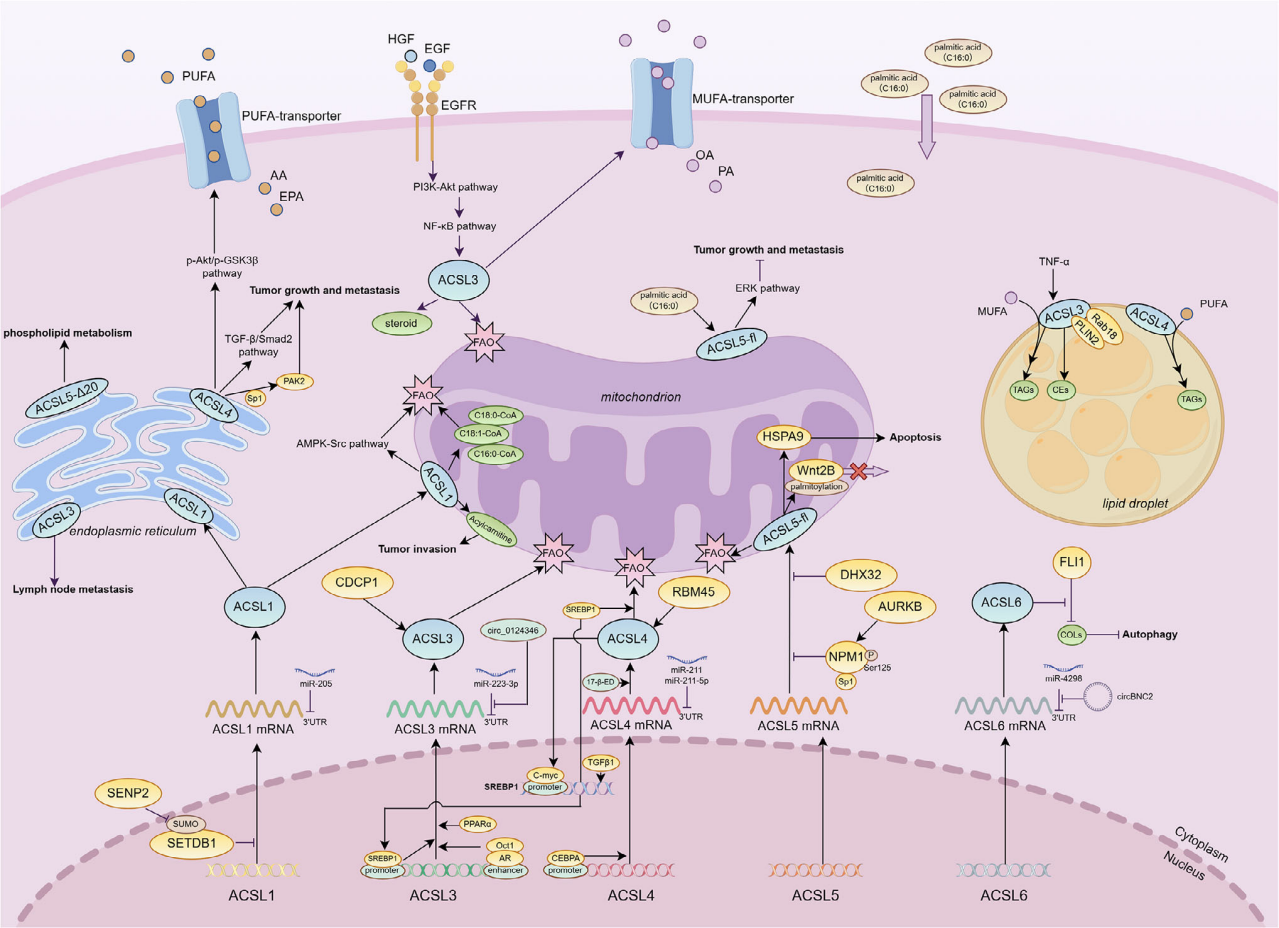
Members of the acyl‐CoA synthetase long‐chain (ACSL) family regulate lipid metabolism reprogramming in tumour cells through different mechanisms. The expression of ACSL family members in tumour cells is regulated at the transcriptional or post‐transcriptional level, and their activity is strictly controlled by many molecules. They can participate in lipid metabolism reprogramming of tumour cells as lipid metabolism‐related enzymes, or activate downstream‐related pathways to regulate the occurrence and development of tumour cells.

The prognostic and therapeutic implications of ACSL1 are underscored by its pan‐cancer clinical relevance. Multi‐institutional cohorts revealed that elevated ACSL1 expression in breast cancer correlates with poor overall survival, diminished anti‐tumour immune infiltration and therapeutic resistance to conventional treatments.^42^ In prostate cancer, ACSL1 promotes C16:0‐, C18:0‐ and C18:1‐CoA synthesis to increase mitochondrial β‐oxidation efficiency, generating ATP to fuel metastasis through enhanced cell motility.^43^ CRC exhibits ACSL1‐driven acylcarnitine accumulation, which enhances invasiveness by modulating integrin signalling—a phenotype conserved across aggressive malignancies.^44^, ^45^, ^46^ The diagnostic utility of ACSL1 extends to cervical cancer and isocitrate dehydrogenase 1 (IDH1)‐mutant gliomas, where ACSL1 overexpression predicts adverse clinical outcomes and disease recurrence.^15^, ^47^ These findings collectively establish ACSL1 as a master regulator of oncogenic lipid remodelling, with targeted pharmacological inhibition holding significant translational promise for metabolic therapy (Figure 2).

### 2.2 ACSL1 drives immunosuppressive microenvironment formation

Macrophage polarisation reprogramming constitutes a central immunological mechanism by which ACSL1 actively fosters tumour immunosuppression through spatiotemporal metabolic regulation. ACSL1 reprograms lipid metabolism in tumour‐associated macrophages (TAMs) by enhancing long‐chain fatty acid activation and directing acyl‐CoA towards phospholipid synthesis. This biases TAM differentiation into pro‐tumourigenic M2 phenotypes via altered eicosanoid signalling and membrane fluidity. In prostate cancer, ACSL1‐enriched TAMs potently suppress CD8^+^ T‐cell infiltration and cytotoxicity by secreting anti‐inflammatory cytokines (IL‐10, TGF‐β) while expressing arginase‐1, correlating significantly with chemotherapy resistance and immune evasion pathways.^26^ HCC models further reveal that ACSL1 suppresses the fatty acid transporter CD36 and key β‐oxidation enzymes, exacerbating intracellular lipid accumulation and disrupting mitochondrial energetics. This metabolic shift concurrently inhibits autophagic flux and oxidative metabolism through activation of the thioredoxin‐interacting protein (TXNIP)/PRKAA (AMPKα)/mechanistic target of rapamycin kinase complex 1 (MTORC1) signalling axis, thereby reinforcing M2 polarisation and creating a self‐sustaining immunosuppressive niche.^48^ Similarly, ACSL1 activates the glucagon‐like peptide 1 receptor (GLP‐1R)/peroxisome proliferator‐activated receptor γ (PPARγ) pathway to suppress pro‐inflammatory M1 polarisation markers (inducible nitric oxide synthase[iNOS], tumour necrosis factor‐α [TNF‐α]) while inducing alternative activation signatures, establishing an immunotolerant tumour microenvironment (TME).^49^

T‐cell exclusion and functional impairment further characterise ACSL1‐mediated immunosuppression through chemokine network dysregulation. In triple‐negative breast cancer (TNBC), ACSL1 activates the MAPK/NF‐κB cascade to secrete GM‐CSF(Granulocyte‐Macrophage Colony‐Stimulating Factor), which drives pro‐inflammatory monocyte differentiation and PD‐L1 upregulation via JAK/STAT signalling. This dual mechanism forms physical and immunological barriers, inhibiting cytotoxic T‐cell activity and inducing T‐cell exhaustion.^50^ Clinically, high ACSL1 expression correlates strongly with elevated tumour immune dysfunction and exclusion (TIDE) scores in breast cancer prognostic models, reflecting impaired T‐cell infiltration, functional exhaustion signatures (TIM‐3, LAG‐3) and diminished cytolytic activity.^51^ Glioblastoma multiforme (GBM) exhibiting ACSL1 overexpression manifests metabolic subtype MC1‐associated immune evasion, characterised by reduced T‐cell infiltration scores and enriched myeloid‐derived suppressor cell (MDSC) populations within the peritumoural space.^52^

Neutrophil modulation and immune checkpoint engagement extend ACSL1's immunosuppressive repertoire through lipid mediator networks. In gastric cancer, ACSL1 orchestrates CD44‐high neutrophil subsets to physically block T‐cell infiltration via CXCL1/CXCL8 secretion while amplifying oxidative stress through NADPH oxidase activation, consequently diminishing anti‐PD‐1 immunotherapy sensitivity and promoting neutrophil extracellular trap (NET) formation.^53^ Conversely, downregulation of ACSL1 in clear cell renal cell carcinoma (ccRCC) is associated with increased expression of immune checkpoints including programmed cell death‐1 (PD‐1, encoded by PDCD1) and cytotoxic T lymphocyte‐associated antigen‐4 (CTLA4), as well as elevated Tumour Immune Dysfunction and Rejection (TIDE) scores, which collectively correlates with impaired anti‐tumour immunity in this malignancy.^54^

Epigenetic dysregulation reinforces ACSL1's role in low‐grade gliomas (LGGs), where promoter DNA hypomethylation drives ACSL1 overexpression. This epigenetic alteration associates with upregulated immune checkpoint (PD‐1/CTLA4) expression and profound T‐cell exhaustion through altered histone modifications at effector gene loci, cementing an immunosuppressive TME resistant to checkpoint blockade.^28^

**FIGURE 3:**
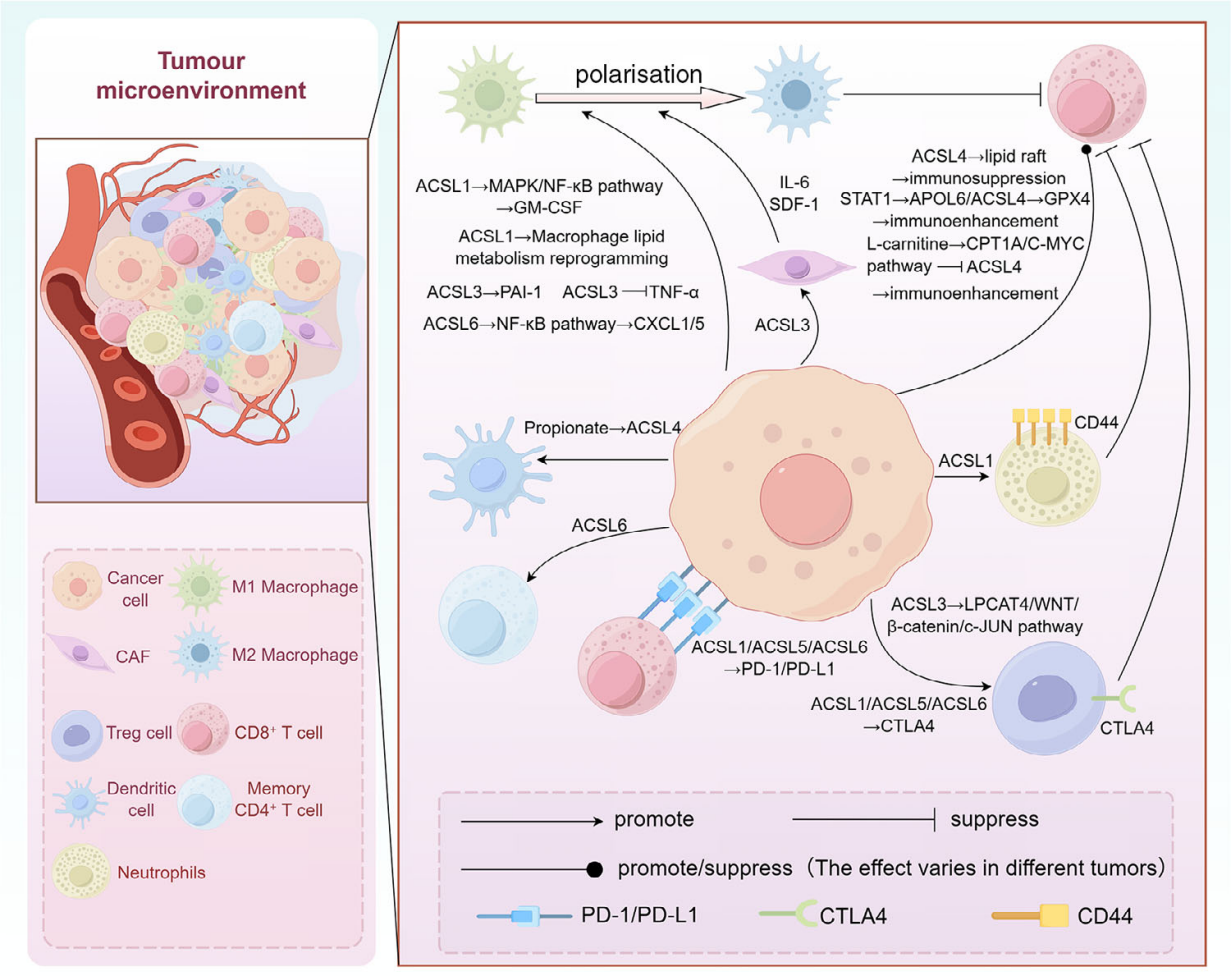
Changes in the expression of acyl‐CoA synthetase long‐chain (ACSL) family members in tumour cells can lead to different effects on some immune cells in the immune microenvironment. Members of the ACSL family regulate the production of inflammatory factors, metabolites or surface receptors by tumour cells, affecting the function of some immune cells in the tumour immune microenvironment.

Collectively, ACSL1 orchestrates multifaceted immunosuppression through integrated metabolic‐immune circuits involving macrophage/neutrophil phenotypic switching, T‐cell dysfunction and checkpoint activation. Its position upstream of PD‐1/CTLA4 regulation and myeloid cell education positions ACSL1 as a promising master regulatory node for immunotherapeutic targeting. Pharmacological inhibition or epigenetic modulation of ACSL1 may reverse T‐cell exclusion and overcome immune checkpoint blockade (ICB) resistance across malignancies, providing combinatorial strategies to enhance existing immunotherapies (Figure 3).

### 2.3 ACSL1‐targeted combination therapy with PD‐1/PD‐L1 inhibitors: Translational potential

ACSL1‐mediated immunosuppression (e.g., M2 macrophage polarisation, impaired CD8^+^ T‐cell infiltration and IL‐10/TGF‐β enrichment) creates an ‘immune desert/excluded’ microenvironment that compromises PD‐1/PD‐L1 checkpoint blockade efficacy.^26^, ^50^, ^54^ Notably, ACSL1 inhibition synergises with PD‐1/PD‐L1 inhibitors through a complementary mechanism: ACSL1 targeting reverses metabolic‐driven immunosuppression to restore T‐cell infiltration, while checkpoint blockers directly relieve T‐cell exhaustion—forming a ‘two‐pronged’ strategy to overcome immune escape.

This combination is most promising for tumours with the molecular signature ACSL1 high expression + PD‐1/PD‐L1 positive + immunosuppressive TIME, including TNBC where ACSL1 overexpression correlates with elevated PD‐L1 and regulatory T‐cell (Treg) infiltration, and ACSL1 inhibition enhances CD8^+^ T‐cell recruitment to sensitise tumours to anti‐PD‐1 therapy^50^; prostate cancer where ACSL1‐driven M2 polarisation suppresses CD8^+^ T‐cell cytotoxicity, with preclinical evidence confirming ACSL1 silencing combined with anti‐PD‐1 antibody exerts tumour‐suppressive effects^26^; ccRCC where ACSL1 high‐expression tumours exhibit reversible immunosuppression (unlike ACSL1 low‐expression tumours with inherent PD‐1/CTLA4 overexpression^54^), rendering them responsive to combined targeting; and LGGs where ACSL1 overexpression (via promoter hypomethylation) associates with PD‐1/CTLA4 upregulation and T‐cell exhaustion, making ACSL1 inhibition plus anti‐PD‐L1 therapy a potential approach to reverse central nervous system immune privilege.^28^

Systematic evidence further supports this synergy: ACSL1 high‐expression tumours rely on metabolic reprogramming rather than irreversible immune exhaustion to establish immunosuppression, providing a molecular basis for combination therapy^54^; PD‐1 inhibitors alone fail to abrogate ACSL1‐mediated M2 polarisation via the GLP‐1R/PPARγ pathway^49^; and ACSL1 silencing restores effector T‐cell function (enhanced IFN‐γ/granzyme B secretion) in LGG.^28^

Collectively, these findings underscore that ACSL1 inhibition is a rational strategy to augment PD‐1/PD‐L1 blockade in specific tumour subtypes, bridging metabolic reprogramming and immune modulation to address unmet clinical needs in immunotherapy‐resistant malignancies.

## 3 ACSL3

Acyl‐CoA synthetase long‐chain family member 3 (ACSL3) exhibits prominent expression across multiple human organs critical for metabolic and secretory functions, including the liver, pancreas, prostate, cardiomyocytes, mammary epithelial cells and vascular endothelial cells.^55^, ^56^, ^57^, ^58^, ^59^, ^60^ Within the cytoplasmic compartment, ACSL3 primarily localises to the ER and lipid droplets (LDs), demonstrating stimulus‐responsive subcellular redistribution dynamics. Upon fatty acid stimulation, it undergoes rapid translocation from the ER membrane to the surfaces of nascent LDs during active lipogenesis, facilitating lipid storage organelle maturation.^61^, ^62^ By enzymatically activating long‐chain fatty acids into acyl‐CoA thioesters, ACSL3 drives the biosynthesis and esterification of neutral lipids, particularly triacylglycerols (TAGs) and cholesteryl esters (CEs), thereby directly promoting LD biogenesis, expansion and cytoplasmic lipid storage capacity.^57^, ^63^ Furthermore, ACSL3 forms essential functional complexes with perilipin‐2 (PLIN2, a major LD scaffold protein) and the small GTPase Rab18. These complexes spatiotemporally coordinate LD growth, stabilisation and fatty acid metabolic flux through targeted substrate channeling.^61^, ^63^

Notably, TNF‐α, a pivotal pro‐inflammatory cytokine in tumour immunology and microenvironment remodelling, transcriptionally upregulates ACSL3 expression. This induction significantly increases LD formation in vascular endothelial cells, mechanistically linking inflammatory signalling cascades to pathological lipid metabolic reprogramming within the tumour vasculature.^58^ Additionally, ACSL3 confers robust cellular resistance to oxidative stress by suppressing membrane lipid peroxidation: it preferentially promotes the incorporation of monounsaturated fatty acids (MUFAs) into membrane phospholipids, thereby indirectly reducing the relative proportion of peroxidation‐susceptible polyunsaturated fatty acid (PUFA)‐containing phospholipids. It further modulates key ferroptosis‐related pathways, reinforcing its role in cell death evasion.^64^ These multifaceted mechanisms underscore ACSL3's critical function as a central regulator integrating lipid metabolism, tumour progression dynamics and immunometabolic crosstalk within malignant microenvironments.

### 3.1 ACSL3 promotes tumour growth and metastasis via lipid metabolism

Through coordinated lipid metabolic reprogramming across diverse malignancies, ACSL3 critically drives tumour progression by orchestrating dual pro‐tumourigenic functions. Its capacity to enhance fatty acid activation and de novo lipogenesis—synergising functionally with ACSL4 in HCC to amplify lipid anabolic flux—concurrently suppresses oxidative stress through peroxidation‐resistant lipid pool formation, thereby accelerating hepatocellular carcinogenesis.^14^ In pancreatic ductal adenocarcinoma (PDAC), the oncogenic circ_0124346/miR‐223‐3p regulatory axis elevates ACSL3 expression to stimulate lipid synthesis and tumour cell proliferation.^65^ Meanwhile, its broader antioxidant effect, mediated by the incorporation of phospholipids containing MUFAs, confers resistance to pan‐cancer ferroptosis.^64^

To meet heightened energy demands in KRAS (V‐Ki‐ras2 Kirsten rat sarcoma viral oncogene homolog)‐driven tumours, ACSL3 facilitates FAO for bioenergetic support. Lung adenocarcinoma (LUAD) exploits ACSL3‐mediated FAO to sustain KRAS mutation‐dependent proliferative signalling and biomass production,^66^, ^67^ whereas CRC activates the transforming growth factor‐β (TGFβ1)/sterol regulatory element‐binding protein 1 (SREBP1)/FAO axis, generating ATP and NADPH to fuel epithelial‐to‐mesenchymal transition (EMT) and metastatic dissemination.^68^ In TNBC, CUB‐domain containing protein 1 (CDCP1) redirects fatty acids towards ACSL3‐governed mitochondrial β‐oxidation, overproducing ATP to drive metastatic progression.^11^ Transcriptional integration with core oncogenic networks defines ACSL3's functional versatility. In prostate cancer, the organic cation transporter 1 (Oct1)/androgen receptor (AR) complex directly activates ACSL3 transcription to promote castration‐resistant intratumoural steroidogenesis,^69^, ^70^, ^71^, ^72^ whereas PPARα‐mediated ACSL3 regulation in HCC correlates strongly with poor patient survival.^73^, ^74^ Malignant gliomas exhibit receptor tyrosine kinase (RTK)/Akt pathway crosstalk, where ACSL3 overexpression drives uncontrolled proliferation via glycogen synthase kinase 3β (GSK3β) inactivation.^75^

Clinically, ACSL3 overexpression stratifies high‐risk molecular subtypes, predicting lymph node metastasis in PDAC,^76^, ^77^ therapy resistance in advanced prostate cancer,^69^, ^70^, ^71^, ^72^ and reduced overall survival in non–small‐cell lung cancer (NSCLC) and HCC cohorts.^73^, ^74^, ^78^ It further defines metabolic subtyping in oestrogen receptor (ER)‐negative breast cancer^79^ and predicts radiotherapy responsiveness.^71^

These collective findings position ACSL3 as a central metabolic linchpin, bridging lipid anabolic, catabolic and oncogenic signalling pathways to drive pan‐cancer progression through multifaceted mechanisms (Figure 2).

### 3.2 ACSL3 modulates tumour cell ferroptosis

MUFA‐centred lipid remodelling is a primary, evolutionarily conserved mechanism by which ACSL3 potently suppresses ferroptosis. By preferentially activating and channelling MUFAs—such as oleic acid (OA)—into pathways driving membrane phospholipid synthesis and LD biogenesis, ACSL3 effectively reduces the abundance of peroxidation‐susceptible PUFAs within cellular membranes, thereby diminishing membrane lipid peroxidation, a fundamental biochemical hallmark of ferroptosis execution. This protective role manifests critically in cancer progression; in breast cancer models, ACSL3 integrates exogenous MUFAs into cellular membranes, shielding dormant metastatic cells from ferroptotic elimination, a process strongly correlated with poor clinical outcomes.^80^ Adipocyte‐derived OA within the TME further reinforces this protection through ACSL3‐dependent esterification and membrane incorporation in breast cancer cells.^81^ Melanoma exploits this axis during lymphatic metastasis, where oleate‐rich lymph fluid promotes ACSL3‐mediated MUFA incorporation into tumour cell membranes, inhibiting lipid peroxidation and facilitating the establishment of metastatic niches.^82^ Similarly, renal clear cell carcinoma (ccRCC) critically relies on ACSL3‐driven sequestration of exogenous fatty acids into cytoplasmic LDs, effectively diminishing peroxidative stress and conferring robust resistance to canonical ferroptosis inducers like erastin and RSL3.^83^, ^84^

Epigenetic and post‐transcriptional regulatory mechanisms significantly amplify the antiferroptotic function of ACSL3 across malignancies. N6‐methyladenosine (m6A) RNA modifications play a crucial role in stabilising ACSL3 mRNA transcripts: in CRC, cancer‐associated fibroblasts (CAFs) secrete METTL3‐loaded exosomes that enhance ACSL3 m6A methylation within tumour cells, while METTL7B mediates analogous stabilising effects in bladder cancer contexts.^24^, ^85^ Conversely, FTO‐dependent m6A demethylation in oral squamous cell carcinoma (OSCC) destabilises ACSL3 mRNA, consequently sensitising tumour cells to ferroptosis induction.^86^ Furthermore, the m6A reader protein IGF2BP3 specifically recognises and binds m6A‐modified ACSL3 transcripts in LUAD, significantly increasing their RNA stability to sustain critical cellular antioxidant defenses.^87^

Clinically, elevated ACSL3 expression robustly predicts intrinsic ferroptosis resistance and correlates with poor prognosis across diverse malignancies, including HCC, lung cancer, cholangiocarcinoma (CCA), prostate cancer and multiple myeloma.^84^, ^88^, ^89^, ^90^, ^91^, ^92^ Its dual significance as both a prognostic biomarker and a promising therapeutic target is further underscored by functional links to key signalling pathways; for instance, in pancreatic stellate cells, IL15RA‐STAT3 signalling drives coordinated ACSL3 and glutathione peroxidase 4 (GPX4) upregulation, fostering a therapy‐resistant phenotype within the TME.^93^

**FIGURE 4:**
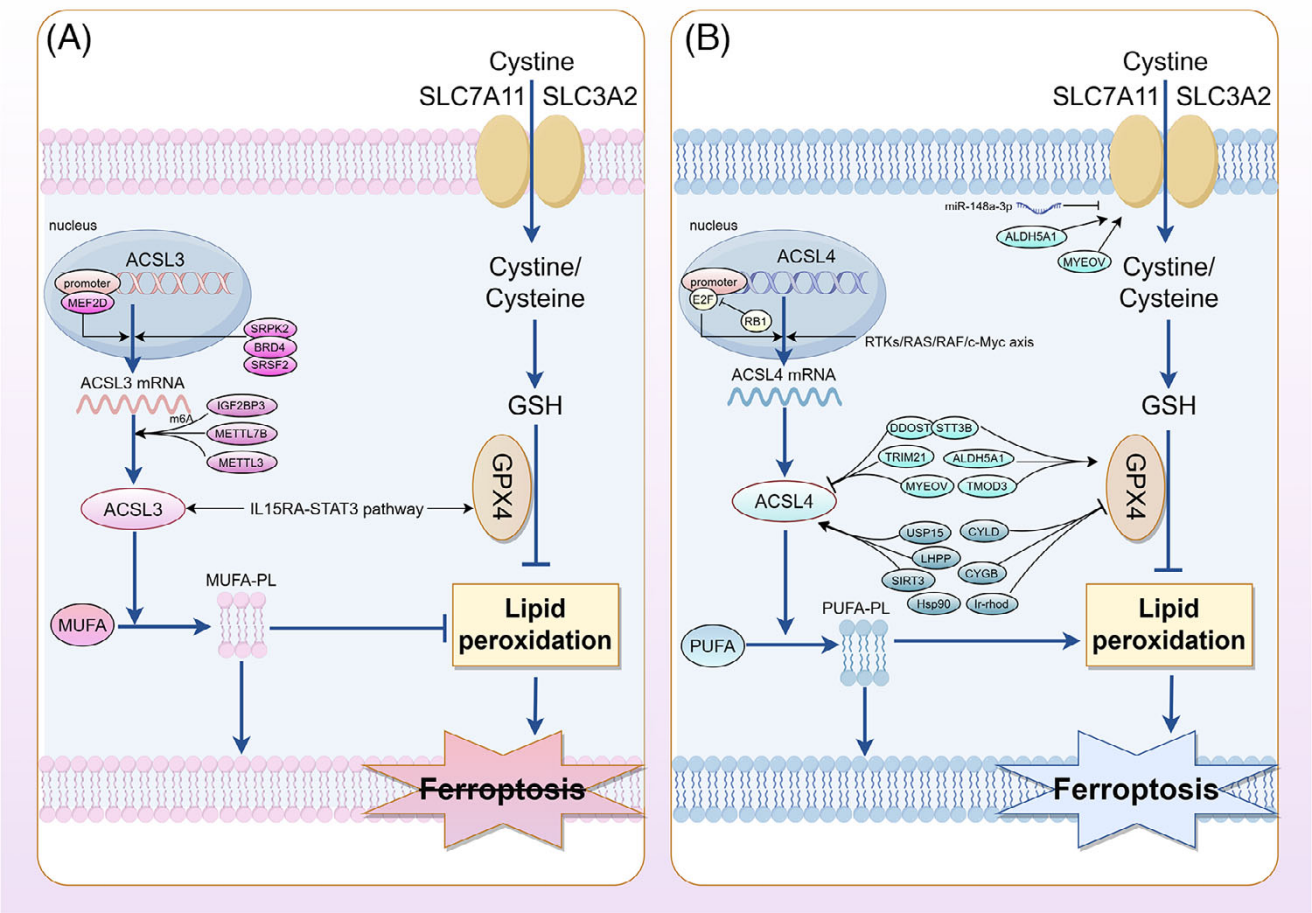
The regulatory mechanisms of acyl‐CoA synthetase long‐chain family member 3 (ACSL3) and acyl‐CoA synthetase long‐chain family member 4 (ACSL4) in ferroptosis of tumour cells. (A) ACSL3 promotes the integration of monounsaturated fatty acids into phospholipid membranes, reduces membrane phospholipid peroxidation levels and synergistically inhibits tumour cell ferroptosis with glutathione peroxidase 4 (GPX4). (B) ACSL4 promotes the integration of polyunsaturated fatty acids into phospholipid membranes, induces lipid peroxidation and leads to ferroptosis of tumour cells.

Overall, ACSL3 orchestrates a comprehensive ferroptosis‐suppressive network spanning lipid metabolic reprogramming, epitranscriptomic regulation and transcriptional control, positioning it as a pivotal therapeutic target for overcoming intrinsic and acquired treatment resistance in multiple cancer types (Figure 4).

### 3.3 ACSL3 facilitates tumour immune evasion

Metabolic reprogramming‐driven immunosuppression fundamentally underpins the role of ACSL3 in facilitating tumour immune evasion in HCC. Mechanistically, lysophosphatidylcholine acyltransferase 4 (LPCAT4) regulates ACSL3 expression via the WNT/β‐catenin/c‐JUN signalling axis, and this LPCAT4‐ACSL3 regulatory cascade enhances de novo cholesterol synthesis.^94^ Additionally, ACSL3 is closely associated with the recruitment of immunosuppressive regulatory T cells (Tregs) into the TIME, which contributes to dampened anti‐tumour immune surveillance.^95^ Consistently, multiple studies have validated ACSL3 as a prognostic‐related biomarker in HCC, further supporting its functional relevance in tumour progression.^95^, ^96^, ^97^ Similarly, in PDAC, ACSL3 mediates the induction of plasminogen activator inhibitor‐1 (PAI‐1), which is closely associated with extensive fibrotic stromal remodelling and increased infiltration of TAMs (predominantly M2‐like subtype). Concomitantly, ACSL3‐PAI‐1 axis activation correlates with suppressed cytotoxic T lymphocyte (CTL) activity and the establishment of an immune‐excluded phenotype, with genetic ablation of either ACSL3 or PAI‐1 shown to reduce fibrosis and TAM infiltration while enhancing CTL function.^98^

Immune checkpoint molecule dysregulation further amplifies ACSL3‐dependent immune escape mechanisms. In LUAD, ACSL3 suppresses IFN‐α secretion to reduce CTL infiltration. It concurrently upregulates ferroptosis‐inhibitory genes (CDGSH iron sulphur domain 1 [CISD1] and 6‐phosphogluconate dehydrogenase [PGD]) via the lncRNA GSEC/miR‐101‐3p axis, weakening immunogenic cell death (ICD) signals.^89^, ^92^, ^99^, ^100^, ^101^, ^102^, ^103^, ^104^, ^105^ Moreover, ACSL3 expression is significantly correlated with aberrant levels of immunosuppressive molecules such as programmed death‐ligand 2 (PD‐L2) and CD96, alongside methyltransferase‐like 14 (METTL14)‐mediated N6‐methyladenosine (m6A) RNA modifications, which collectively drive acquired resistance to ICB therapies.^89^, ^101^

Complex epigenetic and transcriptional crosstalk intricately links ACSL3 activity to dynamic tumour–immune interactions. Prostate cancer exemplifies this interplay, where ACSL3 enhances EMT, amplifies TGF‐β signalling cascades and modifies extracellular matrix interactions to directly inhibit anti‐tumour immunity. It concurrently stimulates interleukin‐6 (IL‐6) and stromal cell‐derived factor 1 (SDF‐1) secretion within CAFs, fostering a radioresistant TIME.^71^, ^72^, ^88^ In ovarian cancer models, ACSL3‐mediated depletion of B cells and cytotoxic lymphocytes correlates strongly with poor patient prognosis and dysfunctional anti‐tumour immunity.^106^

Paradoxical roles for ACSL3 within the immune context are notably observed in ccRCC, where its downregulation, often associated with specific DNA methylation aberrations, leads to reduced immune cell infiltration and profound lipid metabolic dysfunction, paradoxically accelerating disease progression by creating a permissive microenvironment.^107^, ^108^ This stark duality underscores ACSL3's highly context‐dependent immunomodulatory functions across different malignancies.

Clinically, ACSL3 has emerged as a significant pan‐cancer orchestrator of immune evasion, operating through multifaceted mechanisms including ferroptosis suppression, oncogenic lipid metabolic rewiring and immune checkpoint activation. Its substantial therapeutic potential is further underscored by functional links to PPAR/angiopoietin‐like 4 (ANGPTL4)‐mediated genomic instability in LUAD^102^ and STAT3‐driven stromal‐immune crosstalk,^93^ positioning ACSL3 as a compelling multimechanistic target for intervention across diverse malignancies (Figure 3).

## 4 ACSL4

The functional versatility of acyl‐CoA synthetase long‐chain family member 4 (ACSL4) is fundamentally governed by its distinct tissue‐specific expression patterns and precise subcellular compartmentalisation. ACSL4 demonstrates particularly high expression within key physiological sites including the steroidogenic adrenal cortex, the metabolically active liver, immune‐responsive microglia and various components of the reproductive system.^109^, ^110^, ^111^, ^112^ Furthermore, ACSL4 exists as multiple alternatively spliced isoforms, each characterised by unique subcellular targeting signals that direct their localisation to specific organelles—notably the ER for phospholipid synthesis, LDs for neutral lipid storage and plasma membrane microvilli for localised signalling events. This strategic compartmentalisation critically governs spatially restricted phospholipid metabolism, enabling ACSL4 to perform niche‐specific functions based on its cellular address and the available lipid precursors.^113^ ACSL4's enzymatic specialisation in activating long‐chain PUFAs, particularly ω6 species like arachidonic acid (AA), to form acyl‐CoA thioesters underpins its dualistic roles in essential membrane biogenesis and ferroptosis regulation. By preferentially utilising these PUFAs, ACSL4 channels activated substrates into pathways driving the synthesis of critical signalling phospholipids, such as phosphatidylinositols.^113^ Concurrently, this identical enzymatic function establishes ACSL4 as a decisive gatekeeper of ferroptotic susceptibility. Its enrichment of peroxidation‐vulnerable PUFAs, primarily arachidonoyl and adrenoyl moieties, into membrane phospholipid pools dramatically amplifies their propensity for oxidative damage and subsequent lipid peroxidation cascades. This process synergises potently with inhibition of the key antioxidant enzyme GPX4, thereby facilitating the lethal accumulation of lipid peroxides that execute iron‐dependent ferroptotic cell death.^114^, ^115^ Significant therapeutic implications emerge directly from ACSL4's inherent mechanistic duality. While its activation of PUFAs can fuel oncogenic lipid remodelling processes driving tumour progression in certain malignancies, ACSL4's potent capacity to promote ferroptosis simultaneously renders it a compelling, context‐dependent therapeutic target. Pharmacological strategies designed to exploit this delicate balance—either through selective ACSL4 inhibition to disrupt pro‐tumourigenic lipid signalling or by enhancing tumour cell sensitivity to ACSL4‐dependent ferroptosis induction in vulnerable contexts—hold considerable promise for advancing precision cancer therapies.^111^, ^115^

### 4.1 Context‐dependent dual roles of ACSL4 in tumourigenesis

The functional duality inherent to ACSL4 critically defines its complex and context‐dependent role in cancer biology, fundamentally balancing pro‐ and anti‐tumourigenic effects through distinct and often opposing mechanistic axes. On one axis, ACSL4's potentiation of ferroptosis, primarily driven by its specific enrichment of PUFAs, particularly ω6‐PUFAs like AA and adrenic acid (AdA), into membrane phospholipids, underpins its significant tumour‐suppressive potential. This enzymatic activity markedly amplifies the susceptibility of these PUFA‐containing phospholipids to peroxidation, a defining biochemical hallmark of ferroptosis execution. Consequently, ACSL4 expression effectively sensitises susceptible tumour cells to oxidative, iron‐dependent cell death triggered by various inducers, thereby positioning ACSL4 as a crucial metabolic vulnerability factor, especially within malignancies heavily reliant on maintaining stringent redox homeostasis for survival.^116^ Conversely, within alternative oncogenic contexts, the dominant activity of ACSL4 shifts towards facilitating profound lipid metabolic rewiring that fuels tumour progression. Here, ACSL4 activates key proliferative and survival signalling cascades, including the potent Akt/GSK3β axis, the master metabolic regulator c‐Myc/SREBP1 pathway and the TGF‐β/SMAD family member 2 (Smad2) signalling cascade, collectively driving extensive lipogenic reprogramming. These ACSL4‐mediated pathways critically fuel malignant phenotypes such as uncontrolled tumour cell proliferation, enhanced invasive capacity and metastatic dissemination, achieved through mechanisms involving the robust enhancement of lipid anabolism and the active promotion of EMT progression.^117^, ^118^, ^119^ These ACSL4‐mediated pathways critically fuel malignant phenotypes such as uncontrolled tumour cell proliferation, enhanced invasive capacity and metastatic dissemination, achieved through mechanisms involving the robust enhancement of lipid anabolism and the active promotion of EMT progression. Thus, ACSL4 exemplifies a sophisticated metabolic rheostat, dynamically modulating cell fate decisions in cancer. Consequently, the successful therapeutic exploitation of ACSL4's duality necessitates the precise stratification of tumours based on the dominant pathway activity to effectively target either its ferroptotic vulnerability or oncogenic lipid dependency.

#### 4.1.1 ACSL4 as an oncogenic driver: Mechanisms and pathways

Lipid metabolic reprogramming constitutes a central oncogenic mechanism underlying ACSL4's pro‐tumourigenic functionality, with distinct pathway activations across malignancies. In HCC, ACSL4 transcriptionally activates the c‐Myc/SREBP1 axis to drive de novo lipogenesis, resulting in pathological accumulation of triglycerides, CEs and LDs; clinical analyses confirm this co‐expression profile correlates with vascular invasion and reduced overall survival.^118^ RNA‐binding motif protein 45 (RBM45) amplifies this metabolic rewiring by directly binding ACSL1/ACSL4 and Rictor mRNAs, coordinating lipogenic gene expression and lipolytic enzyme suppression to promote HCC progression through lipid storage expansion and β‐oxidation inhibition.^120^ Sp1‐mediated transcriptional activation of the ACSL4‐PAK2 axis similarly promotes HCC growth and metastasis via Rac1/Cdc42 cytoskeletal remodelling, while tumour‐suppressive miR‐211‐5p silences ACSL4 to impair FAK/Src signalling and inhibit invasive potential.^121^, ^122^ Chronic liver injury models further reveal ACSL4's role in exacerbating fibrogenesis and post‐transcatheter arterial chemoembolisation (TACE) recurrence through C/EBPα‐driven mitochondrial β‐oxidation adaptation under glucose deprivation, promoting chemoresistance through ATP maintenance.^25^, ^123^

Hormonal signalling integration critically defines ACSL4's oncogenicity in oestrogen receptor α‐positive (ERα+) breast cancer. 17β‐Estradiol‐activated ERα directly upregulates ACSL4 expression to increase PUFA uptake—including AA and eicosapentaenoic acid—driving migration, proliferation and EMT through E‐cadherin transcriptional suppression and p‐Akt/p‐GSK3β/β‐catenin signalling cascade activation.^117^

Metastatic adaptation mechanisms further underscore ACSL4's multifaceted oncogenicity. Ovarian carcinoma cells exploit ACSL4‐mediated phospholipid remodelling to increase membrane fluidity and invadopodia formation, sustaining peritoneal metastatic outgrowth via ABHD6/ECI1/ECH1‐mediated FAO that fuels ATP‐dependent invasion machinery.^124^ In osteosarcoma, TGF‐β/Smad2‐dependent ACSL4 transcriptional activation promotes proliferation and migration through fibronectin–integrin signalling reinforcement, which is pharmacologically reversible upon ACSL4 silencing.^119^

These interconnected mechanisms collectively position ACSL4 as a molecular linchpin of oncogenic lipid remodelling across malignancies, with tissue‐specific pathway dominance dictating precision therapeutic strategies targeting lipid metabolic vulnerabilities (Figure 2).

#### 4.1.2 ACSL4 as a tumour suppressor: Ferroptosis‐driven mechanisms

Ferroptosis induction fundamentally underpins the tumour‐suppressive functionality of ACSL4 across diverse malignancies through lipid peroxidation cascade amplification and membrane destabilisation mechanisms. In CRC, miR‐148a‐3p directly targets the cystine/glutamate antiporter SLC7A11 to relieve its inhibitory effect on ACSL4, triggering extensive phospholipid peroxidation and ferroptosis‐mediated tumour growth arrest via mitochondrial voltage‐dependent anion channel activation and cardiolipin externalisation.^125^ Multilayered post‐translational regulatory mechanisms further modulate this axis: the oligosaccharyltransferase complex DDOST/STT3B orchestrates site‐specific N‐glycosylation of cathepsin D (CTSD) at asparagine 263, modulating its tertiary structure and proteolytic cleavage efficiency towards acyl‐CoA dehydrogenase medium chain (ACADM), which subsequently regulates ACSL4 palmitoylation status to impact CRC metastatic invasiveness through altered membrane fluidity and integrin clustering dynamics.^126^ Cytoglobin (CYGB) enhances ferroptosis sensitivity via the tumour protein p53‐Yes‐associated protein 1 (p53‐YAP1) transcriptional cascade that upregulates ACSL4 expression and AA incorporation, whereas KRAS‐mutant tumours exhibiting ACSL4 promoter hypermethylation demonstrate profound ferroptosis resistance and adverse clinical outcomes due to impaired PUFA metabolism and enhanced glutathione recycling capacity.^127^, ^128^

Hippo/YAP and AKT signalling pathway crosstalk defines context‐dependent ACSL4 regulation through phosphorylation‐dependent degradation switches and transcriptional networks. In metastatic castration‐resistant prostate cancer, deubiquitinase cylindromatosis (CYLD) stabilises YAP by inhibiting β‐TrCP‐mediated K48‐linked ubiquitination, consequently upregulating ACSL4 and transferrin receptor (TFRC) to amplify lipid peroxidation through enhanced iron uptake and preferential incorporation of oxidation‐prone fatty acids into mitochondrial phospholipids.^129^ Currently, tumour suppressor phospholysine phosphohistidine inorganic pyrophosphate phosphatase (LHPP) blocks AKT/SKP2‐mediated ACSL4 phosphorylation at serine 575, preventing its CRL4 E3 ligase recognition and proteasomal degradation, thereby prolonging pro‐ferroptotic activity through sustained arachidonoyl‐CoA synthesis and peroxidation cascade propagation.^130^ RB1‐deficient prostate adenocarcinomas exploit E2F‐driven ACSL4 transcriptional activation via promoter demethylation to increase ferroptosis susceptibility, creating a therapeutic vulnerability for GPX4 inhibitors through synthetic lethality in homologous recombination‐defective tumours characterised by BRCAness phenotypes.^131^, ^132^

Therapeutic resistance paradigms and molecular intervention strategies highlight ACSL4's pivotal clinical relevance across neuro‐oncology and solid tumours. Glioblastomas with ACSL4 epigenetic silencing resist radiotherapy due to impaired radiation‐induced lipid peroxidation and failed mitochondrial permeability transition, whereas heat shock protein 90 (Hsp90) stabilises ACSL4 via PP2A‐mediated dynamin‐related protein 1 (Drp1) Ser637 dephosphorylation to promote mitochondrial fission‐coupled lipid peroxidation through cardiolipin externalisation and cytochrome c release amplification.^29^, ^133^ Additional innovative interventions targeting ACSL4‐regulated ferroptosis pathways will be systematically detailed in subsequent methodology‐focused sections covering nanoparticle delivery and metallodrug strategies.

Unique regulatory circuits further diversify ACSL4's pathophysiological roles through oncogene cooperation and dietary modulation. Superenhancer‐driven oncogene MYEOV in EGFR‐mutant LUAD stabilises the ACSL4/GPX4 protein complex via HSP70 chaperone activity to constitutively block ferroptosis execution and promote anoikis resistance,^134^ whereas high‐fat diets transcriptionally suppress ACSL4 via PPARδ‐SREBP1 activation cascades to promote EMT and metastatic invasion through LD biogenesis and TGF‐β pathway potentiation.^135^, ^136^ Melanoma leverages constitutive RTK/RAS/RAF/c‐Myc signalling to transcriptionally activate ACSL4 through AP‐1 binding sites, enhancing erastin sensitivity through heightened arachidonate‐containing phospholipid biosynthesis and altered redox homeostasis.^137^ Sirtuin 3 (SIRT3)‐mediated AKT inhibition in gallbladder carcinoma upregulates ACSL4 via FOXO3a nuclear translocation and HDAC4 sequestration, suppressing EMT through ferroptosis‐dependent degradation of Snail transcription factor and ZEB1 protein destabilisation.^138^

Overall, ACSL4 serves as a master ferroptosis rheostat integrating metabolic inputs, genetic vulnerabilities and microenvironmental cues, with its therapeutic targeting potential maximised in tumours exhibiting heightened PUFA synthesis, impaired glutathione regeneration, defective phospholipid repair mechanisms or mitochondrial fragmentation predisposition—positioning it as a cornerstone molecular target for next‐generation precision oncology approaches exploiting lipid metabolic dependencies (Figure 4).

### 4.2 ACSL4 modulates tumour–immune crosstalk

The regulation of ICD positions ACSL4 as a context‐dependent dual modulator of tumour immunogenicity through lipid metabolic rewiring. In melanoma, ACSL4 enriches PUFAs in phospholipids to remodel membrane lipids and form lipid rafts via ceramide‐sphingomyelin domains. This structure blunts platinum‐induced immunogenic ferroptosis and pyroptosis by sequestering death receptors and blocking gasdermin pore formation. Pharmacological disruption of these specialised membrane microdomains enhances mitochondrial permeability transition‐dependent ICD, improves CD8^+^ T‐cell effector function through enhanced antigen cross‐presentation, and potentiates systemic anti‐tumour immunity by amplifying dendritic cell maturation.^16^ Conversely, in acute myeloid leukaemia (AML), the microbial metabolite propionate induces ACSL4‐dependent ferroptosis via PINK1/Parkin‐mediated mitophagy, increasing leukaemia cell immunogenicity through calreticulin exposure and ATP release, thereby promoting dendritic cell activation and antigen‐specific T‐cell priming for robust anti‐tumour responses.^139^ A key mechanism of CD8^+^ T cell (CTL)‐mediated tumour killing involves interferon‐γ (IFN‐γ) derived from CTLs, which in combination with AA triggers ACSL4‐dependent immunogenic ferroptosis in tumour cells.^140^ Mechanistically, IFNγ upregulates ACSL4 to alter tumour cell lipid profiles, facilitating the incorporation of AA into C16 and C18 acyl chain‐containing phospholipids, while circulating palmitoleic acid and OA further enhance this ferroptotic process.^140^ Clinically, tumour ACSL4 expression correlates with T‐cell signature enrichment and improved survival in patients receiving ICB.^140^ In contrast, in glioblastoma (GBM), ACSL4 contributes to pro‐tumourigenic necrosis by mediating neutrophil‐triggered ferroptosis: tumour‐infiltrating neutrophils transfer myeloperoxidase (MPO)‐containing granules into tumour cells, inducing iron‐dependent lipid peroxide accumulation, and ACSL4 depletion reduces GBM necrosis and aggressiveness.^141^ This context‐dependent role of ACSL4—either promoting anti‐tumour immunity or facilitating tumour progression—highlights its complex regulation of tumour–immune crosstalk.

The immune checkpoint interplay further defines ACSL4's therapeutic relevance within metabolic‐immunological networks. In NSCLC, the carnitine palmitoyltransferase 1A (CPT1A)/c‐Myc feedback loop transcriptionally suppresses ferroptosis by downregulating ACSL4 expression while concurrently activating the nuclear factor erythroid 2‐related factor 2 (NRF2)/GPX4 antioxidant axis through Keap1 degradation. Targeted CPT1A inhibition restores ACSL4‐mediated lipid peroxidation through mitochondrial reactive oxygen species (ROS) accumulation, synergising with ICB by reversing T‐cell exhaustion and enhancing tumour immunogenicity.^142^ In muscle‐invasive bladder cancer, ACSL4 interacts with STAT1 to transcriptionally activate APOL6. APOL6 forms mitochondrial pores to amplify ferroptosis, enhancing PD‐L1 blockade efficacy by promoting CTL infiltration and reducing MDSC accumulation.^30^ In HCC, eukaryotic translation initiation factor 3 subunit f (eIF3f) stabilises ACSL4 via K48‐linked deubiquitination, promoting fatty acid biosynthesis; the subsequent accumulation of fatty acids in the TME indirectly impairs CD8^+^ T‐cell infiltration and activation, while targeting the eIF3f‐ACSL4 axis enhances the efficacy of anti‐PD‐1 therapy.^143^

Collectively, ACSL4 serves as a pivotal metabolic–immune interface where its contextual modulation of ferroptotic susceptibility fine‐tunes anti‐tumour immunosurveillance. This is achieved through diverse mechanisms, including regulation of CTL‐mediated ferroptosis, neutrophil‐induced tumour necrosis, and metabolic suppression of T‐cell infiltration—all of which are exploitable for next‐generation immunotherapies. Combinatorial strategies that simultaneously target ACSL4‐dependent lipid metabolism and immune checkpoint evasion mechanisms hold great promise for improving clinical outcomes in cancer patients (Figure 3).

## 5 ACSL5

The intricate functional complexity of ACSL5 is defined by its tissue‐selective expression profile and evolutionarily conserved isoform diversity, establishing context‐dependent roles in cellular metabolism and survival regulation. ACSL5 demonstrates predominant expression in intestinal crypt epithelia, hepatocyte populations, renal tubular cells and antigen‐presenting immune cells including dendritic cells and macrophages,^144^, ^145^, ^146^, ^147^ with its subcellular compartmentalisation dictated by alternative splicing events: the canonical full‐length isoform (ACSL5‐fl) localises to mitochondrial outer membranes where it regulates cristae architecture and apoptosis initiation, while the truncated splice variant ACSL5‐Δ20 anchors to ER membranes to modulate phospholipid acyl chain remodelling.^145^ This spatial segregation underpins ACSL5's dual functionality in coordinating apoptosis execution and lipid homeostasis. ACSL5‐fl governs intrinsic apoptosis through acyl‐CoA‐dependent cardiolipin peroxidation that facilitates BAX/BAK oligomerisation and cytochrome c release in hepatocytes and intestinal epithelia,^144^ whereas ACSL5‐Δ20 orchestrates ER phospholipid saturation states, impacting membrane fluidity, autophagosome biogenesis and calcium signalling fidelity through phosphatidylinositol metabolism.^148^ These compartmentalised functions integrate via dynamic crosstalk with oncogenic signalling cascades: ACSL5‐derived lipid metabolites activate Wnt/β‐catenin proliferative pathways while concurrently suppressing STAT3‐mediated inflammatory responses, thereby mechanistically linking membrane lipid composition to proliferation‐inflammation equilibrium.^147^, ^149^

A well‐recognised function of ACSL5 is its regulation of FAO: as a key enzyme catalysing the activation of long‐chain fatty acids into fatty acyl‐CoA esters,^149^ ACSL5‐fl supplies critical substrates for mitochondrial FAO [Reinartz et al., 2010] integrating lipid metabolism with energy production to sustain cellular homeostasis. This FAO‐regulatory role intersects with its apoptotic and metabolic functions, as dysregulated ACSL5‐mediated FAO contributes to lipoapoptosis in metabolically stressed cells.^146^, ^147^

Critical clinical implications emerge from this functional duality and FAO‐regulatory capacity. While ACSL5‐fl's proapoptotic activity and FAO‐modulating function suggest tumour‐suppressive potential in epithelial malignancies by limiting metabolic adaptation, ACSL5‐Δ20's metabolic rewiring promotes chemoresistance through LD accumulation and may drive malignancy progression by altering FAO substrate availability and membrane receptor dynamics.^145^ This functional antagonism, coupled with ACSL5's central role in FAO‐dependent energy metabolism, positions isoform‐specific therapeutic targeting as a promising strategy to exploit ACSL5's context‐dependent vulnerabilities across cancer types.

### 5.1 Context‐dependent dual roles of ACSL5 in tumourigenesis

ACSL5 exhibits profound tumour type‐specific functional duality, operating as either an oncogenic driver or tumour suppressor through context‐dependent modulation of lipid metabolic reprogramming, apoptotic resistance pathways and intricate signalling network crosstalk. This dichotomous behaviour reflects tissue‐specific molecular landscapes that determine its ultimate impact on carcinogenesis.

Pro‐tumourigenic mechanisms predominate in malignancies where ACSL5 drives metabolic adaptation favouring proliferation. In gastric adenocarcinoma, one cut homeobox 2 (ONECUT2)‐mediated promoter hypomethylation epigenetically upregulates ACSL5 expression, accelerating intestinal metaplasia‐to‐carcinoma progression through enhanced AA incorporation into membrane phospholipids; conversely, pharmacological ACSL5 inhibition significantly suppresses xenograft tumourigenicity by disrupting prostaglandin E2 synthesis.^150^ In luminal breast carcinomas, ACSL5 downregulation attenuates lipogenic gene expression (including FASN and HMGCS1), limiting acetyl‐CoA availability for energy production and membrane biogenesis, thereby restricting tumour growth through metabolic constraint.^151^

Tumour‐suppressive functions dominate alternative contexts through distinct molecular pathways. CRC involves ACSL5‐mediated apoptotic sensitisation via mitochondrial HSPA9 chaperone regulation, a conserved mechanism functionally linked to *TP53* status that promotes cytochrome c release; DEAH‐box helicase 32 (DHX32) overexpression antagonises this axis by disrupting HSPA9‐ACSL5 interaction, consequently promoting proliferation and metastatic dissemination.^152^, ^153^ During intestinal carcinogenesis, ACSL5 cooperates with Porcupine acyltransferase to catalyse Wnt2B palmitoylation, restricting its nuclear translocation through enhanced membrane sequestration and suppressing β‐catenin‐driven proliferative signalling.^154^, ^155^ LUAD models further demonstrate ACSL5‐mediated growth inhibition through palmitic acid (C16:0)‐induced ceramide accumulation, coupled with enhanced major histocompatibility complex class I (MHC‐I) antigen presentation via lipid raft reorganisation, synergistically potentiating PD‐1 blockade efficacy through improved neoantigen recognition.^156^, ^157^

Collectively, ACSL5's functional pleiotropy—spanning membrane lipid metabolism, apoptotic resistance modulation and immune checkpoint regulation—highlights its context‐dependent therapeutic potential as either a target for inhibition or reactivation, necessitating precision approaches tailored to tumour‐specific molecular environments (Figure 2).

### 5.2 ACSL5 orchestrates tumour–immune crosstalk

CD8^+^ T‐cell functional modulation underscores ACSL5's context‐dependent immunoregulatory duality across malignancies. In LUAD, OSCC, cutaneous melanoma, and osteosarcoma, ACSL5 enhances CD8^+^ T‐cell cytotoxicity through mitochondrial membrane lipid remodelling, promotes tumour infiltration via CXCL10 chemokine induction, and synergises with PD‐1 blockade to suppress tumour progression through enhanced immune synapse formation.^158^, ^159^, ^160^, ^161^ Conversely, immunosuppressive niches emerge in hormone receptor‐positive breast carcinomas, where elevated ACSL5 expression correlates with CD8^+^ T‐cell functional inhibition and reduced intratumoural infiltration through PGE2‐mediated exhaustion pathways. As a peroxisome‐related gene within validated prognostic risk models, ACSL5 further predicts diminished response to immune checkpoint inhibitors through STAT3‐mediated immunosuppressive programming.^27^ Similarly, ACSL5 overexpression in PDAC and high‐grade gliomas associates with mesenchymal transition phenotypes, poor survival outcomes and immune desertification characterised by absent T‐cell infiltrates.^28^, ^162^

Beyond T‐cell‐centric regulation, ACSL5 influences broader immunological landscapes through lineage‐specific mechanisms. During LUAD precursor progression, ACSL5 upregulation coincides with B‐cell activation through CD40 signalling and NK cell suppression via reduced NKG2D ligand expression, suggesting compartmentalised immunomodulation across lymphocyte subsets.^163^ This immune polarisation directly aligns with therapeutic resistance: melanoma patients with ACSL5‐high tumours exhibit elevated PD‐1/CTLA‐4 co‐expression on tumour‐infiltrating lymphocytes and attenuated response to checkpoint blockade through Treg‐mediated suppression.^164^ In CRC, ACSL5 serves as a core component of fatty acid metabolism gene signatures, where elevated metabolic activity scores predict unfavourable outcomes and primary immunotherapy refractoriness through SREBP1‐dependent LD accumulation.^165^

Mechanistically, ACSL5 remodels membrane lipid metabolism to dictate immune cell recruitment and functional states. By altering phospholipid acyl chain composition and eicosanoid precursor availability, it modulates dendritic cell antigen presentation capacity through enhanced MHC‐I trafficking^158^ and regulates checkpoint ligand expression via NF‐κB‐mediated transcriptional control. These integrated metabolic–immune interfaces position ACSL5 as a therapeutically tunable target to overcome resistance to PD‐1/CTLA‐4 inhibitors through combinatorial strategies that simultaneously disrupt lipid‐mediated immunosuppression and enhance T‐cell effector function (Figure 3).

## 6 ACSL6

Tissue‐selective expression patterns and neurological prominence fundamentally define the biological niche of ACSL6, with predominant localisation observed in cerebral tissues, bone marrow microenvironments and dopaminergic neuronal populations within the substantia nigra and ventral tegmental areas.^166^, ^167^, ^168^, ^169^ This compartmentalised distribution facilitates ACSL6's enzymatic catalysis of long‐chain fatty acids—notably docosahexaenoic acid (DHA)—into acyl‐CoA derivatives, positioning it as a master regulator of neural lipid metabolism governing synaptic membrane composition and myelin integrity through selective esterification pathways.

Neural structural integrity and functional specialisation constitute hallmarks of ACSL6 activity within the central nervous system. By preferentially enriching DHA‐containing phospholipids in neuronal membranes, ACSL6 maintains axonal structural stability and modulates dopaminergic neurotransmission efficiency, critically influencing motor coordination, reward circuitry signalling and photoperiod adaptation through retinal dopamine metabolism modulation.^166^, ^167^ Its functional purview extends to synaptic vesicle cycling and neurodevelopmental processes, where perturbations in ACSL6‐mediated lipid acyl chain remodelling correlate with synaptic pruning defects and are mechanistically linked to neurodevelopmental disorders including autism spectrum conditions and dopaminergic dysfunction pathologies.

Diverse regulatory networks govern ACSL6's functional versatility across physiological contexts. Insulin‐dependent transcriptional regulation via FOXO1 signalling fine‐tunes ACSL6 expression in metabolic tissues during fasting–feeding transitions,^170^ while intersecting DHA metabolic cascades and inflammatory signalling pathways (e.g., NF‐κB transcriptional cascades activated by TNF‐α) cooperatively shape cellular stress responses and membrane repair mechanisms.^171^ Intriguingly, ACSL6 engages with ferroptosis execution pathways through modulation of PUFA incorporation into phospholipid pools, suggesting dual roles in redox homeostasis maintenance and iron‐dependent cell death susceptibility within oxidative stress microenvironments.^172^

Oncogenic and immunomodulatory implications emerge from ACSL6 dysregulation across pathologies. In haematological malignancies, recurrent chromosomal translocations generating ETV6::ACSL6 fusion proteins constitutively activate JAK‐STAT signalling cascades, driving leukaemogenic proliferation and therapy resistance in myeloid neoplasms.^173^, ^174^ Beyond haematopoiesis, ACSL6 modulates tumour–immune interactions through biosynthesis of immunoregulatory lipid mediators (resolvins, protectins), thereby influencing inflammatory microenvironment polarisation, dendritic cell maturation states and immune checkpoint molecule expression patterns including PD‐L1 transcriptional regulation.^175^

Overall, ACSL6 functionally interconnects neural lipid metabolism, oncogenic transformation and immune regulation networks, offering multipronged therapeutic entry points for targeting neurological disorders and neoplastic diseases through precision modulation of its tissue‐specific lipid remodelling activities.

### 6.1 ACSL6 in cancer: Mechanistic insights and clinical implications

Chromosomal aberrations and haematologic malignancies constitute principal manifestations of ACSL6's oncogenic involvement, with specific translocations driving leukaemogenesis through dysregulated cytokine signalling. The recurrent t(5;12)(q31;p13) translocation generates an oncogenic ETV6::ACSL6 fusion protein that transcriptionally activates interleukin‐3 (IL‐3) overexpression, promoting eosinophil clonal expansion in AML and acute lymphoblastic leukaemia (ALL); this molecular aberration correlates strongly with treatment resistance, disease relapse and diminished overall survival outcomes.^169^, ^171^, ^173^, ^174^ In chronic myeloid leukaemia (CML), ACSL6 serves as a ferroptosis‐related diagnostic biomarker reflecting profound iron metabolism dysregulation, where its expression patterns mirror aberrant iron accumulation and glutathione depletion characteristic of blast crisis progression.^175^

Prognostic biomarker duality underscores ACSL6's context‐dependent roles across epithelial malignancies. CRC and HCC exhibit ACSL6 overexpression correlated with advanced TNM staging, EMT and significantly reduced progression‐free survival, positioning it as an adverse prognostic indicator through promotion of metastatic dissemination.^97^, ^176^, ^177^, ^178^, ^179^, ^180^, ^181^ Paradoxically, in prostate adenocarcinoma, activation of the circBNC2/miR‐4298/ACSL6 regulatory axis stimulates lipid peroxidation cascades that promote ferroptotic cell death, consequently inhibiting tumour progression through iron‐dependent cytotoxicity.^172^ TNBC further illustrates this contextual duality: patients with elevated ACSL6 expression demonstrate favourable therapeutic responses characterised by enriched memory CD4^+^ T‐cell infiltration within tertiary lymphoid structures, reduced M2‐polarised macrophage populations and improved long‐term survival outcomes, highlighting its immunomodulatory potential within specific TMEs.^182^

Ferroptosis induction and immune crosstalk mechanistically define ACSL6's multifaceted functionality. While its pro‐ferroptotic activity in prostate cancer restrains tumourigenesis through peroxidation of PUFAs in phospholipid membranes, ACSL6 simultaneously modulates lipid‐mediated immune cell recruitment in TNBC by altering eicosanoid precursor availability, thereby enhancing CD4^+^ T‐cell chemotaxis and activation. This functional dichotomy illustrates profound metabolic‐immunological interplay wherein membrane lipid composition dynamically regulates both cell death susceptibility and anti‐tumour immunity (Figure 2).

### 6.2 ACSL6 modulates antigen presentation and immune cell function

The intricate interplay between ACSL6 and immune checkpoint pathways critically defines its role in sculpting therapeutic responsiveness across malignancies. In AML, elevated ACSL6 expression correlates with elevated ACSL6 expression correlates with enhanced CD160 molecule (CD160, a co‐stimulatory molecule) presentation and IFN‐γ pathway activation, consequently modulating sensitivity to immune checkpoint inhibitors by augmenting T‐cell‐mediated tumour recognition.^183^ CML exploits the ACSL6‐ferroptosis axis to sustain an immunosuppressive niche through altered lipid peroxidation signalling; conversely, pharmacological ferroptosis induction disrupts this protective microenvironment and resensitises therapy‐resistant CML to conventional chemotherapeutic agents.^175^

Cytokine‐driven immunomodulatory mechanisms mediated by ACSL6 emerge prominently in solid tumours. In HCC, ACSL6 activates the IL‐18R1/NF‐κB/CXCL1/5 cascade to recruit tumour‐associated neutrophils and M2 macrophages, while suppressing CD8^+^ T‐cell infiltration via chemokine dysregulation to mediate immune evasion.^184^ Functioning as a pro‐ferroptotic regulator, ACSL6 further exacerbates HCC immunosuppression by molecularly coupling ferroptosis resistance with coordinated upregulation of PD‐L1 and CTLA‐4 immune checkpoints, establishing dual barriers to immunotherapeutic intervention.^178^, ^179^, ^185^

Immune cell polarisation governed by ACSL6 reveals profound context‐dependent outcomes. TNBC exhibits improved immunotherapy responsiveness in ACSL6‐high tumours, characterised by enrichment of memory CD4^+^ T‐cell populations, reduction of immunosuppressive M2 macrophage infiltration and enhanced tertiary lymphoid structure formation.^182^ Conversely, CRC exploits ACSL6 to transcriptionally upregulate PD‐L1/CTLA‐4 expression through NF‐κB‐mediated pathways, effectively blunting checkpoint blockade efficacy by reinforcing T‐cell exhaustion programs.^185^

Lipid metabolic rewiring fundamentally underpins ACSL6‐mediated immunometabolic crosstalk through biosynthesis regulation of immunomodulatory lipid mediators. By altering the production of prostaglandins (e.g., PGE2), resolvins and specialised pro‐resolving mediators, ACSL6 fine‐tunes dendritic cell antigen‐presenting capacity, modulates macrophage polarisation states, and regulates T‐cell activation thresholds across diverse TMEs, thereby integrating metabolic and immunological circuits.

Collectively, ACSL6 orchestrates context‐specific immune evasion or activation through multifaceted mechanisms: NF‐κB signalling pathway manipulation, ferroptosis sensitivity regulation and metabolic‐immune axis integration. This functional plasticity positions ACSL6 as a therapeutically tunable target for rational combination immunotherapies designed to overcome resistance barriers and restore anti‐tumour immunity (Figure 3).

## 7 INTERPLAY AMONG METABOLIC REPROGRAMMING, FERROPTOSIS AND TUMOUR IMMUNE MICROENVIRONMENT REMODELLING: A MULTI‐DIMENSIONAL REGULATORY NETWORK

The biological functions of the ACSL family in cancer are not isolated but form a complex and interconnected regulatory network involving metabolic reprogramming, ferroptosis and TIME remodelling, which collectively dictates tumour progression and therapeutic response. Metabolic reprogramming, centred on ACSL‐mediated fatty acid activation, serves as the foundational link integrating the three processes: ACSL isoforms selectively channel long‐chain fatty acids into lipid synthesis or β‐oxidation, providing both biosynthetic precursors for membrane biogenesis and energy for tumour cell proliferation, while simultaneously shaping ferroptosis susceptibility and immune cell function through lipid metabolite remodelling.^186^

ACSL‐mediated metabolic rewiring directly modulates ferroptosis sensitivity: ACSL4 promotes the incorporation of PUFAs into membrane phospholipids, enhancing lipid peroxidation and ferroptosis, whereas ACSL3 favours MUFA activation to suppress ferroptosis.^116^, ^125^ This metabolic regulation of ferroptosis further interfaces with TIME remodelling: ferroptotic tumour cells release immunogenic signals (e.g., ATP, calreticulin) that promote dendritic cell maturation and CD8^+^ T cell recruitment, while ACSL1‐driven M2 macrophage polarisation and ACSL5‐mediated T‐cell function modulation create immunosuppressive or immunostimulatory TIME phenotypes, respectively.^26^, ^113^ Notably, lipid metabolic reprogramming in immune cells—such as ACSL‐dependent FAO in regulatory T cells and M2 macrophages—reinforces immunosuppression, while targeting ACSL isoforms can reverse this metabolic bias to restore anti‐tumour immunity.^186^

Reciprocally, the TIME and ferroptosis feedback to regulate ACSL‐mediated metabolism: pro‐inflammatory cytokines (e.g., IFN‐γ) from effector T cells upregulate ACSL4 expression, amplifying ferroptosis in tumour cells, while hypoxia in the TIME suppresses ACSL4‐dependent lipid peroxidation and enhances ACSL3‐driven metabolic adaptation.^140^, ^187^ This bidirectional crosstalk highlights that ACSL family members act as central nodes in coordinating metabolic‐immune‐cell death circuits, a phenomenon consistent with the critical role of lipid metabolic reprogramming in shaping the immunomodulatory landscape of tumours.^186^ Targeting this integrated network—for example, combining ACSL4 agonists with immune checkpoint inhibitors or ferroptosis inducers—holds great promise for overcoming therapeutic resistance by simultaneously disrupting metabolic adaptation, enhancing ICD, and reversing immunosuppression.

## 8 ACSL FAMILY MEDIATES MULTIFACETED MECHANISMS OF TUMOUR DRUG RESISTANCE

Members of the ACSL family orchestrate complex multidrug resistance networks across diverse malignancies through multifaceted pathophysiological mechanisms centred on ferroptosis suppression, fatty acid metabolic reprogramming and autophagy dysregulation, establishing them as critical determinants of therapeutic failure through their master regulatory roles in lipid‐mediated adaptation pathways.

Ferroptosis suppression constitutes a core resistance axis, primarily driven by ACSL4 degradation or functional inhibition. In gastrointestinal stromal tumours, the E3 ubiquitin ligase TRIM21 collaborates with deubiquitinase USP15 to mediate K48‐linked polyubiquitination and proteasomal targeting of ACSL4, facilitating its rapid turnover and conferring imatinib resistance by attenuating lipid peroxidation cascades.^188^ In pancreatic adenocarcinoma, tropomodulin 3 (TMOD3) promotes actin cytoskeletal reorganisation to enhance autophagosome‐lysosome fusion, driving selective autophagic degradation of ACSL4 and inducing resistance to ferroptosis inducers and PD‐1 blockade.^189^ Similarly, aldehyde dehydrogenase 5A1 (ALDH5A1) downregulation in ESCC compromises ACSL4‐mediated PUFA peroxidation, driving cisplatin resistance.^190^

Transcriptional and splicing regulation of ACSL3 further reinforces ferroptosis‐related resistance. In HCC, myocyte enhancer factor 2D (MEF2D) directly transactivates ACSL3 by binding its promoter, blunting sorafenib‐induced ferroptosis through reduced incorporation of peroxidation‐susceptible fatty acids.^191^ In osteosarcoma, bromodomain‐containing protein 4 (BRD4) forms a splicing regulatory complex to enhance ACSL3 pre‐mRNA exon inclusion, amplifying its AA esterification capacity and conferring erastin resistance.^192^

ACSL5 exhibits context‐dependent resistance functions through subcellular compartmentalisation and expression modulation. In rhabdomyosarcoma, aurora kinase B (AURKB) phosphorylates the NPM1/SP1 complex to suppress ACSL5 expression, promoting evasion of mitochondrial apoptosis and GPX4‐regulated ferroptosis.^193^ In GBM, ACSL5 maintains mitochondrial cardiolipin homeostasis and cristae architecture, protecting p53‐deficient neoplasms from Triacsin c‐induced intrinsic apoptosis.^194^, ^195^

Lipid metabolic reprogramming by ACSL isoforms directly enables chemoresistance. ACSL3‐driven biosynthesis of monounsaturated phospholipids in CCA creates a protective membrane composition resistant to RSL3‐induced lipid peroxidation,^84^ while ACSL1‐mediated FAO reprogramming counteracts cisplatin efficacy in TNBC by enhancing NADPH regeneration, ATP production and acetyl‐CoA pools for epigenetic modifications.^196^ Concurrently, ACSL6 modulates therapeutic vulnerability through autophagy pathway hijacking: its FLI1‐collagen axis transcriptionally suppresses radiation‐induced autophagic flux, driving radioresistance in NSCLC by impairing stress‐responsive protein clearance.^197^ Collectively, these interconnected molecular mechanisms establish ACSL isoforms as master regulators of a multi‐dimensional resistance network spanning cell death pathways, metabolic adaptation and stress response systems, positioning them as pivotal therapeutic targets for overcoming oncological treatment barriers through precision intervention strategies targeting lipid metabolic vulnerabilities across cancer types (Table 2).

**TABLE 2:** The resistance mechanisms of different tumours mediated by acyl‐CoA synthetase long‐chain (ACSL) family members.

ACSL family members	Cancer type	Drug resistance mechanism	Citations
•itati	TNBC	ACSL1↑ → FAO↑ → Cisplatin resistance	^196^
•96PER	HCC	MEF2D↑ → ACSL3↑ → Inhibit ferroptosis → Sorafenib resistance	^191^
	OS	ACSL3↑ → AA↑ → Inhibit lipid peroxidation → Erastin resistance	^192^
	CCA	ACSL3↑ → MUFA↑ → Reduce lipid peroxidation → RSL3 resistance	^84^
•4YPER	PC	TMOD3↑ → ACSL4↓ → Inhibit ferroptosis → PD‐1 antibody resistance	^189^
	GIST	TRIM21/USP15↑ → ACSL4↓ → Inhibit ferroptosis → Imatinib resistance	^188^
	ESCC	ALDH5A1↑ → ACSL4↓ → Inhibit lipid peroxidation → Cisplatin resistance	^190^
•90PER	RMS	AURKB↑ → ACSL5↓ → Inhibit apoptosis/ferroptosis → Vincristine resistance	^193^
	GLI	ACSL5↑ → Cardiolipin↑ → Inhibit apoptosis → Etoposide resistance	^195^
•ACSL6	LC	ACSL6↑ → Inhibit cellular autophagy → Radiation resistance	^197^

All citations are derived from studies that experimentally confirmed the causal link between ACSL isoform dysregulation and drug resistance. The resistance mechanism is explicitly demonstrated in the cited literature.

## 9 EMERGING ANTI‐CANCER DRUG DEVELOPMENT TARGETING THE ACSL FAMILY

As extensively documented in preceding sections, the ACSL family emerges as a strategically significant therapeutic target in contemporary oncology owing to its fundamental regulatory functions governing lipid metabolism reprogramming, ferroptotic cell death induction and immunomodulation across diverse malignancies. Current pharmacological development initiatives predominantly concentrate on ACSL4 exploitation, capitalising upon its unique capacity to orchestrate molecular cascades that simultaneously provoke tumour‐selective ferroptosis through iron‐dependent lipid peroxidation mechanisms and activate anti‐tumour immunity via dendritic cell maturation and T‐cell recruitment pathways. Exemplifying this paradigm, mitochondria‐directed iridium complexes demonstrate remarkable efficacy in pulmonary and bladder carcinomas by directly binding ACSL4 gene promoter regions containing antioxidant response elements (AREs) to enhance its transcriptional activity, while concurrently targeting mitochondrial membranes to suppress GPX4 expression. This dual, ACSL4‐specific mechanism cooperatively initiates synchronous ferroptotic and apoptotic pathways that overcome chemoresistance barriers while maintaining favourable toxicity profiles.^198^, ^199^

The marine‐derived alkaloid Lepadins constitutes another promising therapeutic avenue, functioning as a multimodal ferroptosis inducer through direct interaction with ACSL4's catalytic domain to enhance its enzyme activity (facilitating PUFA activation into acyl‐CoA esters), alongside coordinated downregulation of the cystine/glutamate antiporter SLC7A11 and GPX4. Beyond ferroptotic activation, these compounds exhibit complementary anti‐tumour mechanisms including substantial upregulation of tumour suppressor p53 and cyclin‐dependent kinase inhibitor p21, effectively arresting neoplastic proliferation at critical cell cycle checkpoints through G1/S phase blockade. Demonstrated efficacy across various solid tumour models underscores their translational potential, pending structural refinements to augment tumour selectivity and pharmacokinetic properties for clinical advancement.^200^

Nanoparticulate systems represent a transformative technological frontier, synergistically enhancing tumour‐specific delivery while amplifying radio‐chemotherapeutic potency and attenuating systemic exposure. Illustratively, synthetic miR‐211 nanoconjugates effectively silence ACSL4 expression in paediatric medulloblastoma by specifically binding the 3′UTR of ACSL4 mRNA to induce its degradation, significantly impeding tumour progression through dual suppression of proliferative signalling and potentiation of apoptotic cascades.^201^ Crucially, ACSL4 targeting inherently engages immunostimulatory circuits: CTFAP nanoplatforms in mammary carcinomas deliver ACSL4‐selective agonists to directly activate its fatty acid activation function, intensifying ACSL4‐mediated lipid peroxidation while synergising with IFN‐γ secretion to establish self‐reinforcing ferroptosis‐immunotherapy loops.^202^ Parallel strategies employing copper‐based nanoliposomes^203^ and dual‐atom nanozymes^204^ similarly exploit ACSL4's substrate preference for AA, enhancing its peroxidation capacity through nanozyme‐mediated iron ion delivery to the ACSL4‐lipid complex to trigger ICD while stimulating cytotoxic T‐lymphocyte activation and IFN‐γ production. Furthermore, near‐infrared‐absorbing chromophore nanoparticles combined with photothermal ablation and sulphasalazine administration disrupt the xCT‐glutathione axis to initiate ferroptosis, subsequently promoting dendritic cell maturation‐dependent ACSL4 upregulation (via MHC‐I antigen presentation) that amplifies lipid peroxidation cascades in an ACSL4‐specific manner.^205^

**FIGURE 5:**
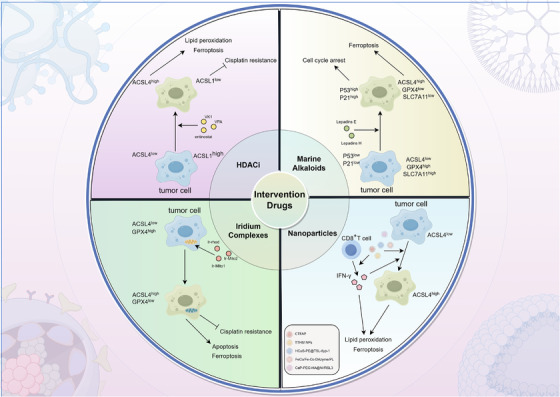
Emerging anti‐tumour drugs targeting the acyl‐CoA synthetase long‐chain (ACSL) family and their mechanisms of action. Four emerging drugs (histone deacetylase [HDAC] inhibitors, marine alkaloids, iridium complexes and nanoparticle coated drugs) can induce ferroptosis in tumour cells by targeting and promoting ACSL4 expression. In addition, HDAC inhibitors and iridium complexes can also contribute to cisplatin sensitisation, respectively, through downregulation of ACSL1 expression or modulation of mitochondrial metabolic pathways. Marine alkaloids can cause tumour cell growth cycle arrest through P53 and P21 sites. Nanoparticle coated drugs can produce anti‐tumour effects by promoting the secretion of IFN‐γ by CD8^+^T cells.

Notably, these immunometabolic mechanisms transcend breast cancer contexts. Biomineralised nanounits in HCC effectively inhibit the STAT3‐PD‐L1 immunosuppressive axis while directly targeting the ACSL4‐PPARγ interaction to enhance ACSL4 expression and lipid peroxidase activity, concurrently improving CD8^+^ T‐cell effector functions to create an immunologically hostile microenvironment for malignant cells.^206^ Intriguingly, epigenetic modulators including histone deacetylase inhibitors (HDACi) demonstrate ACSL‐directed specificity: class I HDACi agents upregulate ACSL4 in gastric malignancies by inhibiting histone deacetylation at the ACSL4 promoter to relieve transcriptional repression and enhance RNA polymerase II binding efficiency, potentiating lipid peroxidation and ferroptosis,^207^ whereas valproic acid reprograms ACSL1‐mediated lipid metabolism by binding ACSL1's AMP‐binding domain to enhance its catalytic activity towards long‐chain fatty acids, reversing cisplatin resistance in TNBC through targeted modulation of the FAO pathway (Figure 5).^196^

Collectively, these multidisciplinary approaches targeting ACSL isoforms—with particular emphasis on ACSL4—underscore the family's indispensable role as central metabolic‐immune signalling nodes governing tumour progression and therapy responsiveness. The convergence of metal complexes, natural products, nanotechnologies and epigenetic modulators, each equipped with ACSL isoform‐specific binding or regulatory mechanisms, establishes a robust therapeutic framework for overcoming oncological challenges through precision modulation of lipid‐mediated cell death pathways, positioning ACSL targeting as a cornerstone strategy in next‐generation cancer therapeutics (Table 3).

**TABLE 3:** Emerging anti‐tumour drugs targeting the acyl‐CoA synthetase long‐chain (ACSL) family and their intervention mechanisms.

ACSL family members	Cancer type	Emerging intervention drugs	Intervention mechanism	Citations
•onsti	TNBC	Valproic acid (VPA)	Reverse ACSL1‐mediated metabolic reprogramming → Reduce cisplatin resistance	^196^
•96PER	MB	miR‐211 nanoparticles	Inhibit ACSL4 → Suppress cell survival and invasion	^201^
	LC	Ir‐rhod	Target mitochondria → Upregulate ACSL4 → Induce ferroptosis	^198^
	GC	VK1, entinostat	Class I HDAC inhibitors → Upregulate ACSL4 → Induce lipid peroxidation and ferroptosis	^207^
	Multiple solid tumours	Lepadins E, H	Upregulate ACSL4/p53 → Activate ferroptosis + G1/S phase arrest	^200^
	BCa	Ir‐Mito1, Ir‐Mito2	Downregulate GPX4 + Upregulate ACSL4 → Synergistically activate ferroptosis and apoptosis	^199^
	BC	CTFAP, TTHM NPs, HCuS‐PE@TSL‐tlyp‐1, FeCo/Fe‐Co DAzyme/PL	Upregulate ACSL4 → Promote ferroptosis + Enhance IFN‐γ secretion by CD8^+^ T cells	^202^, ^203^, ^204^, ^205^
	HCC	CaP‐PEG‐HA@Ni/RSL3	Upregulate ACSL4 + Inhibit STAT3‐PD‐L1 axis → Enhance ferroptosis and CD8^+^ T‐cell function	^206^

The intervention mechanisms of emerging drugs are validated in the cited studies, with citations prioritising those that directly characterise the interaction between the drug and ACSL family members.

## 10 FUTURE PERSPECTIVES OF NOVEL DRUGS TARGETING THE ACSL FAMILY

Novel drugs targeting the ACSL family, including nanoparticle‐based delivery systems and iridium(III) complex photosensitisers, hold substantial translational potential in oncology by leveraging ACSL's core roles in lipid metabolic reprogramming, ferroptosis regulation and tumour–immune crosstalk.^208^, ^209^ However, their clinical advancement is hindered by concise ACSL‐specific limitations: isoform heterogeneity and functional redundancy across malignancies, insufficiently selective targeting systems and TME‐related delivery barriers tied to ACSL‐mediated metabolic complexity.^210^, ^211^, ^212^ Addressing these bottlenecks through precision design will unlock their full clinical value.

The future development of ACSL‐targeted therapies should focus on mechanism‐driven optimisation: engineer isoform‐selective agents such as PROTACs conjugated with ACSL subtype‐unique ligands^213^ and iridium complexes modified with ACSL‐preferred fatty acid derivatives,^214^, ^215^ while developing aptamer‐functionalised or biomimetic nanoparticles for enhanced targeting specificity.^210^, ^216^, ^217^ Harness ACSL‐mediated metabolic‐immune crosstalk via nanocarriers co‐delivering ACSL‐targeted agents with immune checkpoint inhibitors or immunomodulators,^209^, ^218^ and target ACSL‐driven TME remodelling to improve drug penetration.^211^, ^219^ Develop ACSL‐responsive stimuli‐triggered delivery systems utilising pH‐, GSH‐ or MMP‐responsive nanocarriers,^220^, ^221^, ^222^ and optimise iridium complexes with self‐chemiluminescent or hypoxia‐activated properties to bypass TME limitations.^223^, ^224^ Integrate multimodal theranostics for real‐time efficacy monitoring^225^, ^226^ and establish companion diagnostics based on ACSL isoform expression profiles for patient stratification.^212^, ^226^ Additionally, leverage nanoparticle lymphatic recirculation mechanisms to enhance accumulation beyond the EPR effect,^227^ and optimise scalable synthesis and long‐term toxicity assessment focusing on ACSL‐expressing normal tissues.^219^, ^228^

Collectively, focusing on ACSL's unique biological functions—isoform specificity, metabolic‐immune integration and context‐dependent regulation—will drive the development of precision‐targeted therapies. By integrating subtype‐selective design, stimuli‐responsive delivery and theranostic guidance, ACSL‐targeted novel drugs will overcome translational barriers and become cornerstones of precision oncology.^209^, ^229^

## 11 LIMITATIONS AND CONCEPTUAL NOVELTY

While ACSL family members have been implicated in multiple forms of regulated cell death (RCD), including apoptosis, autophagy and pyroptosis,^10^, ^190^, ^224^ this review prioritises ferroptosis, which constitutes a limitation by narrowing the scope of RCD pathways discussed. This strategic focus is justified by three core rationales: First, mechanistic evidence is most robust—ACSL3 and ACSL4 exhibit well‐characterised, opposing roles in ferroptosis regulation (ACSL3 suppresses ferroptosis via MUFA remodelling; ACSL4 promotes ferroptosis through PUFA enrichment) with extensive in vitro and in vivo validation,^64^, ^116^, ^125^ whereas their roles in other RCD pathways are relatively scattered and lack systematic causal validation. Second, translational relevance is higher—ferroptosis inducers (e.g., erastin, RSL3) and ACSL‐targeted agents have advanced to preclinical and early clinical development,^193^, ^195^ and combinatorial strategies (e.g., ACSL4 upregulation + radiotherapy, ACSL3 inhibition + ferroptosis inducers) show clear therapeutic potential,^133^, ^186^ which is less established for ACSL‐mediated apoptosis or autophagy modulation. Third, crosstalk with tumour immunity is more intimate—ferroptosis serves as a key link between metabolic reprogramming and ICD,^139^, ^140^ and ACSL‐mediated ferroptosis regulation directly impacts T‐cell infiltration, macrophage polarisation and immune checkpoint expression,^99^, ^107^ forming a functional network that is the focus of current precision oncology research. This focus does not negate the importance of other RCD pathways but reflects a deliberate choice to deepen insights into the most well‐validated and translationally actionable mechanism.

Recent reviews have explored ACSL family functions from complementary angles, highlighting the conceptual novelty of this work. A broad overview of ACSLs has been provided across diverse diseases (metabolic disorders, cardiovascular diseases, etc.), but this work lacked a focused analysis of cancer‐specific mechanisms and systematic integration of tumour immunity.^230^, ^231^ Another review centred on ACSL‐mediated ferroptosis and anti‐tumour immunity, while emphasising the need for further exploration of ACSL functions in immune cells, without comprehensively dissecting the context‐dependent roles of all five isoforms or providing detailed translational strategies.^232^ Additionally, ACSL‐driven fatty acid metabolism and cell death in cancer have been bridged, but the focus was primarily on metabolic rewiring and ferroptosis crosstalk, with limited coverage of isoform‐specific immunomodulatory mechanisms and emerging drug development.^233^ In contrast, this review offers three distinct conceptual advances: (1) integrated triple‐network framework—it systematically links ACSL‐mediated ‘lipid metabolic reprogramming’ to ‘ferroptosis regulation’ and ‘TIME remodelling’, revealing their interdependence rather than treating them as isolated processes; (2) pan‐family heterogeneity and context dependency—it comprehensively dissects all five ACSL isoforms (ACSL1–6) with detailed analysis of their subtype‐specific, tumour‐type‐dependent roles (e.g., ACSL1 in immunosuppression, ACSL3 in ferroptosis resistance, ACSL4 in ferroptosis promotion), addressing the gap of prior reviews focusing on individual isoforms or broad family functions; (3) translation‐oriented synthesis—it prioritises clinically actionable combinatorial strategies (e.g., ACSL1 inhibition + PD‐1/PD‐L1 blockers, ACSL4 upregulation + radiotherapy) and emerging drug development (e.g., mitochondria‐targeted iridium complexes, ACSL4‐responsive nano‐platforms), bridging basic mechanisms to preclinical/clinical applications with specific tumour subtype stratification.

Finally, a critical limitation that should be emphasised for the clinical translation of ACSL‐targeted anti‐tumour strategies is the constitutive and high expression of ACSL family members in normal tissues, especially the liver, brain and skeletal muscle. ACSL isoforms exert indispensable physiological functions in maintaining lipid metabolism, energy homeostasis and neuronal function in normal organs. For instance, ACSL1 is highly involved in FAO and thermogenesis in hepatic and adipose tissues; ACSL3 and ACSL4 dominates lipid peroxidation and phospholipid synthesis in the liver; and ACSL6 is essential for the maintenance of PUFA‐enriched membranes in neurons of the central nervous system. Therefore, systemic inhibition or global knockout of ACSLs may induce severe off‐target toxicities, including hepatic metabolic disorders, neurotoxicity, impaired motor ability and disrupted energy metabolism, which greatly restrict the clinical application of targeting ACSLs.

To address this obstacle and promote tumour‐specific intervention, several feasible strategies are proposed for future investigation. First, the development of TME‐responsive delivery systems, such as pH‐sensitive, ROS‐sensitive or enzyme‐responsive nanocarriers, can achieve the selective release of ACSL inhibitors in the tumour site while reducing unnecessary exposure in normal liver and brain tissues. Second, the application of tumour‐specific gene editing systems driven by tumour type‐matched promoters represents a precise and translatable approach to restrict ACSL knockout to malignant cells. Specifically, the alpha‐fetoprotein (AFP) promoter can be employed for HCC, as it is exclusively activated in hepatoma cells but silenced in normal hepatocytes; the thyroid transcription factor 1 (TTF‐1) promoter is suitable for LUAD, with specific activation limited to LUAD stem cells; and the mucin 1 (MUC1) promoter is an optimal choice for pancreatic cancer due to its cancer‐specific high expression in pancreatic tumour cells. This design ensures that ACSL depletion is strictly confined to the target tumour type, thereby avoiding the impairment of ACSL physiological functions in normal tissues.^234^ Third, isoform‐selective targeting is another promising direction: designing specific inhibitors for tumour‐enriched ACSL isoforms (e.g., ACSL3 and ACSL6) rather than ubiquitously expressed isoforms can effectively minimise off‐target effects. In addition, local administration or interventional therapy for solid tumours can further reduce systemic toxicity. Collectively, although ACSLs represent promising targets for tumour therapy, the rational design of tissue‐specific and tumour‐selective delivery systems, combined with tumour type‐matched genetic regulation strategies, is essential to translate these findings into safe and effective clinical practice.

Collectively, this review provides a more focused, integrated and translatable perspective on ACSL family functions in cancer, complementing existing literature by synthesising metabolic, cell death and immunological dimensions into a cohesive framework for precision oncology. It is important to introduce the key limitation of off target toxicity in the clinical translation of ACSL‐targeted tumour therapy and propose several feasible solutions, providing new ideas for the clinical translation of this approach.

## 12 CONCLUSION

Lipid metabolic reprogramming represents a pivotal hallmark of malignant transformation and progression, wherein the ACSL family plays a central role by fuelling tumour cell activities through enhanced lipid metabolism. However, as discussed herein, ACSL family members extend beyond lipid metabolism to orchestrate tumour immunity and ferroptosis, exhibiting context‐dependent roles—either pro‐tumourigenic or tumour‐suppressive—across diverse malignancies. We further summarised animal or cell models for knocking out various members of the ACSL family, and summarised the specific effects of knocking out each member in different models (Table 4). Recent advances in immunotherapy have expanded its application in cancers such as HCC, melanoma, NSCLC and CRC. Our analysis revealed that ACSL isoforms are frequently dysregulated in these malignancies and intricately modulate immune cell dynamics within the TIME. These findings position ACSL members as promising therapeutic targets to increase immunotherapy efficacy through isoform‐specific inhibitors or agonists tailored to tumour types. Ferroptosis‐related pathways have emerged as critical determinants of the response to radiotherapy. Radiotherapy eliminates tumour cells via ROS generation, whereas ferroptosis execution hinges on iron accumulation and lipid peroxidation. Notably, combining ferroptosis inducers (e.g., RSL3 and FIN56) with radiotherapy has synergistic effects, particularly in radioresistant tumours.^234^ Given the regulatory roles of ACSL3 and ACSL4 in ferroptosis across malignancies, targeting these isoforms alongside radiotherapy may offer novel therapeutic strategies.

**TABLE 4:** Specific effects of knocking out acyl‐CoA synthetase long‐chain (ACSL) family members in different tissues/organs/cells.

Experiment type	Knockout gene	Knockout model (animal/cell)	Effect	Citations
In vivo experiments	•n viv	Mouse skeletal muscle/adipose tissue	Inhibits fatty acid oxidation	^232^
		Whole‐body knockout in mice	Impairs thermogenic function, reduces cold tolerance and disrupts energy metabolism	^34^
	•4YPER	Whole‐body knockout in mice	Slows down the progression of pancreatic cancer in mice, attenuates tumour fibrosis and alleviates the immunosuppressive microenvironment	^77^, ^98^
	•8YPER	Mouse liver	Inhibits the production of lipid peroxidation products and greatly attenuates liver fibrosis	^25^, ^116^
	•16PER	Whole‐body knockout in mice	Decreases DHA‐enriched phospholipids in neuron membranes by 50%, reduces total PUFA in the striatum; causes hyperkinesia and impairs spatial memory; decreases DHA‐phospholipids in the central nervous system and increases AA‐enriched phospholipids	^166^, ^167^
		Dopaminergic neuron‐specific knockout in mice	Causes hyperkinesia	^167^
		Astrocyte‐specific knockout in mice	Decreases DHA content in the central nervous system, with no change in AA content	^166^
		Lung tissue‐specific conditional knockout in mice	Significantly slows down tumour growth rate, reduces tumour volume and weakens proliferation ability	^197^
In vitro experiment	•n vit	PANC‐1	Reduces lipid droplet accumulation and makes cells more susceptible to autophagy induction	^77^
		B16F10, HEK‐293, A549, HT‐1080	Decreases ferroptosis resistance	^64^, ^187^
	•87PER	HepG2, HL60, LNCaP, Yumm5.2, B16F10, MEFs	Decreases PUFA content or PUFA‐PL synthesis, increases ferroptosis resistance	^16^, ^116^, ^140^, ^187^
		ES‐2‐MC2‐Hep	Decreases cell membrane fluidity and invasion ability, and significantly reduces PUFA content	^124^
		Huh7	Reduces ATP production	^123^
		A549, H460	Inhibits lipid peroxidation and the expression of ferroptosis marker PTGS2	^133^
	•33PER	TDCL, Hepa1–6, A549, SNU387	Downregulates cell‐surface MHC‐I molecule expression; tumour cells resist CD8^+^T cell‐mediated cytotoxicity	^158^
	•58PER	H460, LLC, A549, H1299	Increases apoptosis, raises the proportion of senescent cells, decreases migration ability and downregulates autophagy	^197^

This table lists the in vivo animal models or in vitro cell models involving the knockout of various ACSL family members from the references cited in this article, as well as the specific effects after knocking out each member, and indicates the cited references.

Across diverse malignancies, core ACSL‐mediated pathways exhibit striking conservation alongside tumour‐type‐specific divergence: ACSL1 consistently drives M2 macrophage polarisation to foster immunosuppression in HCC, prostate cancer and TNBC,^26^, ^50^, ^54^ while ACSL3 universally suppresses ferroptosis via MUFA remodelling across CCA, renal clear cell carcinoma and melanoma.^84^, ^87^, ^88^ In contrast, ACSL4 displays context‐dependent duality—promoting ferroptosis as a tumour suppressor in CRC and LUAD,^125^, ^138^ yet functioning as an oncogenic driver via lipid anabolism in HCC and ER+ breast cancer^117^, ^118^—a key controversy that highlights the necessity of isoform‐ and tumour‐type‐specific stratification.^186^ Our synthesis of data identifies the ACSL‐mediated ‘lipid metabolic reprogramming‐ferroptosis‐immune microenvironment’ regulatory axis as the core mechanism governing tumour progression and therapeutic response, with isoform functional heterogeneity being the primary barrier to translational success.

Beyond radiotherapy, drug development targeting the ACSL family (particularly ACSL4) exhibits a diversified landscape. Metal complexes (e.g., iridium‐based agents), natural bioactive molecules (e.g., marine alkaloid Lepadins), nanotechnology platforms and epigenetic modulators (e.g., HDAC inhibitors) demonstrate clinical potential across multiple tumour types. These advances highlight multi‐dimensional solutions to overcome solid tumour treatment barriers: precise intervention in the ACSL4‐mediated ferroptosis‐immunity feedback loop, development of isoform‐selective ACSL inhibitors (e.g., ACSL1/ACSL4), innovative nanodelivery systems for enhanced targeting and combinatorial epigenetic modulation to reverse drug resistance. Translational relevance is further underscored by ongoing clinical investigations: early‐phase trials (e.g., NCT05101889) evaluating ω‐3 PUFA supplementation to enhance ACSL4‐mediated ferroptosis in head and neck cancer,^186^ and phase II studies of class I HDAC inhibitors (e.g., entinostat) combined with anti‐PD‐1 therapy in gastric cancer, leveraging HDACi‐induced ACSL4 upregulation and lipid peroxidation.^204^ These trials validate our central premise that isoform‐selective ACSL targeting, paired with companion diagnostics based on ACSL expression profiles,^140^, ^157^ is the most viable path to clinical benefit.

Future studies should elucidate the spatiotemporal dynamics of ACSL isoforms, optimise drug selectivity and validate synergistic efficacy through clinical translation. From our perspective, resolving the functional redundancy between ACSL isoforms (e.g., ACSL3/ACSL4 in lipid peroxidation regulation)^84^, ^116^ and refining tissue‐specific delivery systems will be critical to overcoming current translational bottlenecks. Pan‐ACSL inhibition is unlikely to succeed due to the family's context‐dependent roles. Subtype specific strategies, such as ACSL4 agonists targeting iron death sensitive tumours and ACSL1/3 inhibitors targeting immunosuppressive or iron death resistant malignant tumours, are more likely to align with the direction of future clinical drug development.

In summary, this review delineates the multifaceted mechanisms by which ACSL family members govern tumour progression, immunity and ferroptosis. These insights provide a robust theoretical foundation for developing precision therapies. Future research should prioritise isoform‐specific drug design and combination regimens to improve the efficacy and safety of cancer treatment.

## AUTHOR CONTRIBUTIONS

Study concept and design: Haocai Li and Weijian Wang. Collecting references: Haocai Li, Juncheng Zhan and Yuxiang Xiao. Original drafting of the manuscript: Haocai Li. Figure preparation and editing: Haocai Li and Chen Su. Writing review and editing: Haocai Li, Weijian Wang and Juncheng Zhan. Project administration: Peng Zhu, Xiaoping Chen and Chen Su. All the authors have reviewed and approved the final version of the manuscript.

## CONFLICT OF INTEREST STATEMENT

The authors declare no conflicts of interest.

## ETHICS STATEMENT

Not applicable.

## Data Availability

No datasets were generated or analysed during the current study.
